# Two-Dimensionality of Yeast Colony Expansion Accompanied by Pattern Formation

**DOI:** 10.1371/journal.pcbi.1003979

**Published:** 2014-12-11

**Authors:** Lin Chen, Javad Noorbakhsh, Rhys M. Adams, Joseph Samaniego-Evans, Germaine Agollah, Dmitry Nevozhay, Jennie Kuzdzal-Fick, Pankaj Mehta, Gábor Balázsi

**Affiliations:** 1 Department of Systems Biology, The University of Texas MD Anderson Cancer Center, Houston, Texas, United States of America; 2 Department of Physics, Metcalf Science Center (SCI), Boston University, Boston, Massachusetts, United States of America; 3 School of Biomedicine, Far Eastern Federal University, Vladivostok, Russia; 4 The Louis and Beatrice Laufer Center for Physical & Quantitative Biology, Stony Brook University, Stony Brook, New York, United States of America; 5 Department of Biomedical Engineering, Stony Brook University, Stony Brook, New York, United States of America; University of Toronto, Canada

## Abstract

Yeasts can form multicellular patterns as they expand on agar plates, a phenotype that requires a functional copy of the *FLO11* gene. Although the biochemical and molecular requirements for such patterns have been examined, the mechanisms underlying their formation are not entirely clear. Here we develop quantitative methods to accurately characterize the size, shape, and surface patterns of yeast colonies for various combinations of agar and sugar concentrations. We combine these measurements with mathematical and physical models and find that *FLO11* gene constrains cells to grow near the agar surface, causing the formation of larger and more irregular colonies that undergo hierarchical wrinkling. Head-to-head competition assays on agar plates indicate that two-dimensional constraint on the expansion of *FLO11 wild type* (*FLO11*) cells confers a fitness advantage over *FLO11 knockout* (*flo11*Δ) cells on the agar surface.

## Author Summary

Microbial biofilms are commonly found in nature and are highly relevant to public health. Biofilms can impose high risks to drinking water distribution by stable adherence to the interior of water pipes, and to food industry by contamination of food processing systems. Biofilm adherence to indwelling medical devices causes high rates of clinical infections that are difficult to eliminate as biofilm microbes resist treatment with antibiotics and biocides. These microbial abilities are related to the spatial composition and overall morphology of the biofilm. While the mechanisms underlying biofilm structure and morphology have been examined for bacteria, much less is known about eukaryotic biofilms. Here we find that the size, shape and patterning of budding yeast colonies can arise from constraining colony expansion to the surface of agar plates. Through computational analysis and mathematical modeling, we find that rapid colony expansion, colony shape irregularity and hierarchical wrinkling of the yeast colony surface can result from two-dimensionality of expansion imposed by the adhesin *FLO11*. Finally, we find that two-dimensional expansion conveys competitive advantage during head-to-head competition with the mutant cells lacking *FLO11*.

## Introduction

Cells often live in large multi-cellular communities. Members of such communities can reproduce and interact with their physicochemical environment, giving rise to complex patterns at spatial scales that greatly exceed the size of individual cells. For example, in embryonic development, complex spatio-temporal patterning results from the interplay of cellular growth, inter-cellular and cell-environmental interactions involving mechanical forces and biochemical signaling [1]. Elucidating the mechanisms of such biological pattern formation is a formidable challenge, which could be aided by understanding pattern formation of simple organisms in well-controlled environments.

Microbial biofilm expansion is among the most important pattern-forming processes. Biofilms are not only ubiquitous in nature, but also occur in hospitals, as the predominant form of contamination on medical devices, such as prostheses and vascular catheters [2]–[5]. Some of these life-threatening infections are difficult to eliminate because biofilms prevent drug access to the constituent microbes [6]. Patterns can develop on biofilm surfaces, as signatures of structures aiding material transport and oxygenation [7]. While the biophysical processes involved in bacterial biofilm formation have been examined [8]–[10], and their evolutionary relevance has been assessed [11]–[13], much less is known about eukaryotic cell communities, despite their relevance to fungal infections [14] and possible implications for cancer [15], [16]. For example, a recent study has implicated localized cell death-mediated buckling in pattern formation during *Bacillus subtilis* biofilm expansion [17], but the generality of these findings across organisms, including eukaryotes, remains to be tested.

The budding yeast, *Saccharomyces cerevisiae*, has emerged as a model organism for studying fungal biofilm formation on solid surfaces [18] or air-liquid interfaces [19]. Common laboratory yeasts have been selected for easy genetic manipulation and handling, and rarely form biofilms. In contrast, many undomesticated or non-standard *S. cerevisiae* variants form highly-patterned biofilms when grown on semisolid agar [18]. The patterns are composed of a central hub region with irregular wrinkles, from which thick wrinkle bundles (spokes) emanate radially towards the peripheral rim [18], [20]. Pattern formation seems to require expression of the surface adhesin *FLO11*, which is under complex environmental regulation, mediated by several transcription factors binding to the largest known yeast promoter, *P_FLO11_* [21]–[24]. In addition, Flo11 protein expression and activity are under post-transcriptional and post-translational control, being targeted to specific subcellular locations, and secreted after cleavage [25]–[30].

Here we seek a unifying mechanism to explain various characteristics of colony expansion that distinguish pattern-forming yeast cells with a functional *FLO11* gene from cells lacking the *FLO11* gene. We accomplish this by comparing quantitative analyses of yeast colony expansion data in well-defined conditions with mathematical and physical models. We find that constrained, two-dimensional colony expansion explains the differences between the two cell types. Moreover, we find that the identified mechanism confers selective advantage during head-to-head competition on agar plates when the two cell types are mixed.

## Results

### The influence of the environment and *FLO11* on colony size

**Figure 1:**
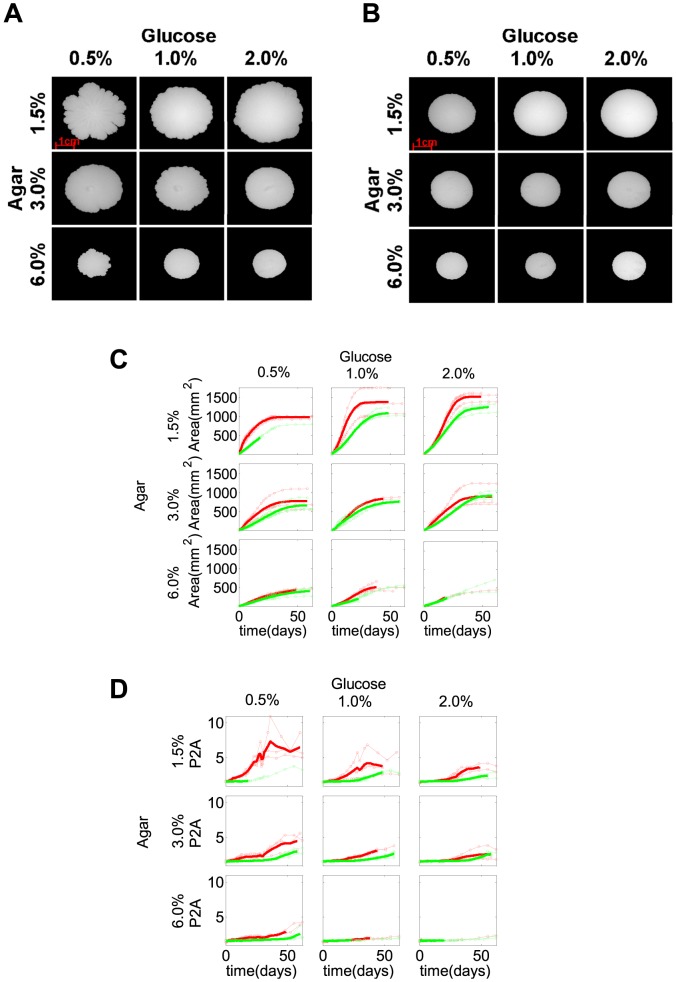
Colony size and irregularity for various glucose and agar concentrations. *(A, B)* Images of *FLO11 (A)* and *flo11Δ (B)* on YPD plates containing different glucose (0.5%, 1.0%, 2.0%) and agar (1.5%, 3.0%, 6.0%) concentrations around day 20. *(C, D)* The expansion of colony area *(C)* and the irregularity *(D)* of *FLO11* (red curves) and *flo11Δ* (green curves) colonies over the 60-day time course. *FLO11* colonies (red curves) demonstrated higher maximum colony size *(C)* with higher irregularity *(D)* at the colony rim than the *flo11Δ* colonies (green curves) in all conditions tested. The maximum colony size *(C)* of both *FLO11* (red curves) and *flo11Δ* (green curves) colonies increased with glucose and inversely depended on agar concentrations. The irregularity of *FLO11 (D)* (red curves) inversely depended on both the agar and the glucose concentrations, compared to the minimal irregularity of *flo11Δ (D)* (green curves) colonies throughout the time course at all conditions tested. Thinner curves represent different replicates while thicker curves represent their average up until all the replicates were present.

We first asked how the presence of a functional *FLO11* gene influenced colony expansion under various nutrient conditions, as previously done in bacteria [31]. To address this question, we applied automated image segmentation followed by pixel counting [32] to obtain the area of *FLO11* and *flo11*Δ colonies that expanded under nine different combinations of agar (1.5%, 3.0%, 6.0%) and glucose (0.5%, 1.0%, 2.0%) concentrations on Yeast Extract-Peptone-Dextrose (YPD) agar plates. We found that *FLO11* colonies (Fig. 1A, C) expanded faster than *flo11*Δ colonies (Fig. 1B, C) and reached larger maximum size in all of these conditions, in agreement with previous observations in soft agar [18]. The maximum colony area increased with glucose concentration, but had an inverse dependence on agar density, regardless of *FLO11* status (Fig. 1C, S1A, B Figure). The time that colonies took to reach their maximum area increased with agar density, with no obvious dependence on glucose concentration, regardless of *FLO11* status (S1E, F Figure). Time derivative of the experimentally measured *FLO11* colony area indicated that the colony expansion curve convexity increased with glucose concentration (S2 Figure). These trends of colony expansion were independent of the sugar type, as suggested by similar trends obtained for agar with galactose (S3 Figure).

We tested whether faster *FLO11* colony expansion could be related to faster growth rate and/or larger cell size of *FLO11* versus *flo11*Δ cells. *FLO11* and *flo11*Δ cells grew at comparable rates in liquid cultures (S4A Figure), and the early expansion rates of *FLO11* and *flo11*Δ microcolonies were similar (S1 and S2 Movies), arguing against a significant difference in their growth rates. Moreover, *FLO11* and *flo11*Δ cells had similar cell size distributions (S4A Figure). Therefore, some other mechanisms must underlie the faster expansion of *FLO11* colonies compared to *flo11*Δ colonies in the same agar and sugar conditions.

### The influence of the environment and *FLO11* on colony shape

The second colony characteristic that we investigated was the colony shape, quantified by analyzing the irregularity of the colony rim (see *Methods*). We used two different methods to quantify rim irregularity, both of which describe the deviation of colony shape from a circle. First, we used the dimensionless P2A ratio, defined as *P* ^2^/4π*A*, where *P* is the perimeter, and *A* is the area of the segmented object. The P2A ratio takes its minimal value of 1 for a perfect circle, and increases as the object becomes more irregular. Second, we calculated the boundary fluctuation (BF) defined as the amplitude of colony edge fluctuations in polar coordinates by using the centroid of the colony as the origin of the new coordinate system (S5 Figure). The P2A method is more sensitive to small fluctuations at the colony rim, whereas BF senses coarser irregularities of colony shape, such as petals and asymmetries. The combination of the two methods quantitatively and comprehensively described the non-circularity at the colony rim.

At each combination of glucose and agar concentrations, both the P2A and BF methods indicated more pronounced colony rim fluctuations in *FLO11* colonies compared to *flo11*Δ colonies (Fig. 1D, S5 Figure). The irregularity at the rim of *FLO11* colonies increased over time for all colonies, suggesting a role for gradual nutrient depletion as described previously for bacteria [31], [33]. The maximum irregularity reached at the end of the time course decreased with both agar and glucose concentrations (Fig. 1D, S5 Figure). The irregularity of the colonies saturated at a much earlier time than the area of the colonies and reached the maximum among all conditions tested, regardless of *FLO11* status (S1 Figure). These trends were consistent between trials and independent of the sugar source.

### A mathematical model captures the effect of sugar concentration on colony expansion

The above measurements indicated that *flo11*Δ colonies tend to be smaller, more circular, with smoother edges compared to *FLO11* colonies (Fig. 1, S5 Figure). Such expansion patterns commonly occur in microorganisms that spread through cell division [34], [35]. Although cells may be non-motile in such colonies, they must move as they divide due to volume limitations. This movement can take place either through spreading on the agar surface or through vertical growth [36]. As long as nutrients are available, and the initial conditions have circular symmetry, a circular colony will grow.

**Figure 2:**
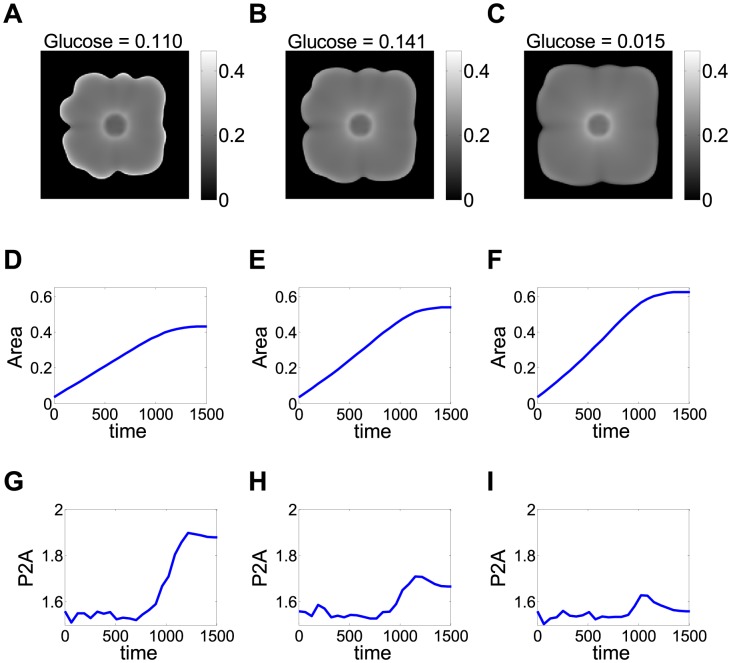
Mathematical model of colony expansion. *(A–C)* A snapshot of the colonies at the end of simulation. Although these simulations are started with circular colonies, over time petals appear. The color scale represents cell density (arbitrary units). *(D–F)* The maximum colony area is higher upon higher initial glucose concentration, in agreement with the experimental results in Fig. 1. The dimensionless “colony area ratio” was the ratio of colony area to the area of simulation box, and glucose concentration corresponded to the initial value of glucose in the simulation, and was chosen as a constant over space. Time is a rescaled variable measured in arbitrary units. *(G–I)* Simulated colony irregularity (P2A) plotted as a function of time. Similar to experiments (Fig. 1), in our model P2A is initially at a basal level and then increases abruptly to a large value. This increase in P2A corresponds to petal formation and occurs as a result of competition over glucose among cells that make up the colony rim. Interestingly, the maximum value of P2A decreases with increasing glucose levels. This result is likely due to decreased intercellular competition over nutrients in the early stages of expansion and is compatible with experiments in Fig. 1, where colonies exhibit less structure as glucose levels increase.

Considering that *FLO11* mediates the adhesion of cells to abiotic surfaces [18], [20], we hypothesized that this causes the cells to stay closer to the agar surface rather than expanding vertically into the third dimension. Therefore, we developed a phenomenological model (see *Methods*) for *FLO11* cells spreading through a diffusive process in two dimensions corresponding to the surface of the agar, similar to earlier studies of colony growth in bacteria [34], [35], [37], [38]. The model's purpose was to identify mechanisms explaining colony size and shape differences, without intending to reproduce exact biological values. Therefore, we applied rescaling to obtain dimensionless variables, such as time and cell density. Cell growth and colony expansion were assumed to be dependent on glucose availability and glucose could diffuse in two dimensions. Using this model, we plotted the time-courses of colony area and irregularity (P2A) for different initial levels of glucose (Fig. 2). The calculated dependence of maximal colony area on environmental parameters (sugar concentration) resembled what was observed experimentally (Fig. 1, S1 Figure). According to the model, the calculated colony area approached its maximum once depleting the available glucose. Furthermore, increasing initial glucose levels in the model lad to larger maximum colony area as in the experiments. Finally, the model also produced a concave curve of colony area versus time at low glucose levels, and a convex curve at high glucose, as in the experiments (Figs. 1, 2 and S2 Supporting Figure). When we modified the model allowing cells to escape into the vertical direction, we obtained smaller colonies (S6 Figure). Overall, the mathematical models supported the hypothesis that *FLO11* yeast colonies tend to grow along the agar surface, which limits their degrees of freedom, leading to larger colony sizes and more irregular colony shapes compared to *flo11*Δ cells that are free to expand vertically.

### Environment- and *FLO11*- dependent hierarchical wrinkling

**Figure 3:**
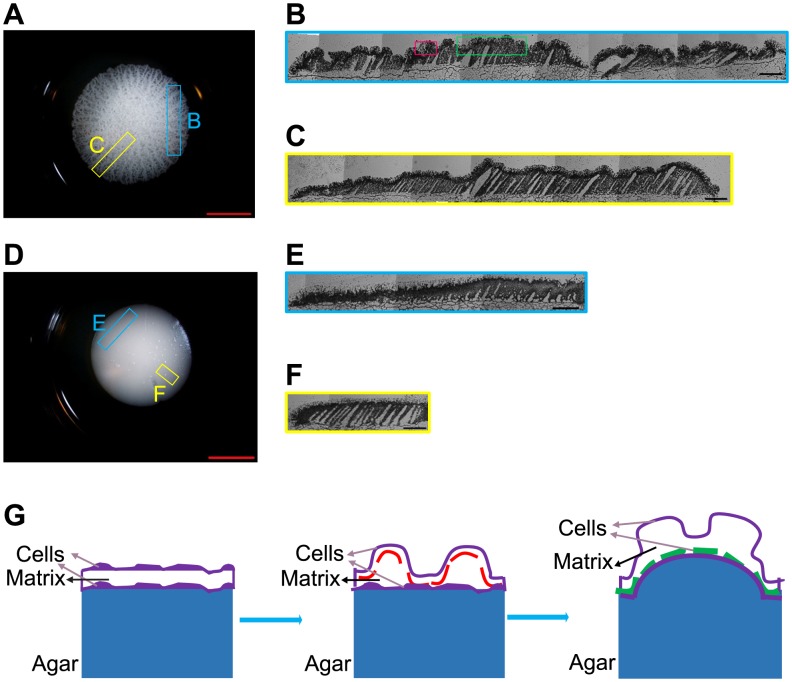
*FLO11*-induced wrinkles on the colony surface. *(A, D)* Frozen blocks oriented across-spokes (blue box indicating estimated location) and radially (yellow box indicating estimated location) were cut from the *FLO11 (A)* or *flo11Δ (D) S. cerevisiae* colonies on 1.5% agar and 1.0% glucose YPD plates. The scale bar was 7500 µm. *(B)* Montage of the cryosections oriented across-spokes of a *FLO11* colony *(A)* indicating wrinkles (red box) and spokes (green box). *(C)* Radial cryosectioning of the *FLO11* colony *(A). (E, F)* Montage of the cryosections oriented across-spokes *(E)* or radially *(F)* for a *flo11Δ* colony *(D)*. The scale bar *(B, C, E, F)* was 500 µm. *(G)* A schematic showing primary wrinkle formation (red dashed line), the saturation of which upon increasing stress leads to secondary wrinkle formation (green dashed line). The agar substrate on which the colony expands is shown in blue.

The third characteristic we investigated was pattern formation on colony surfaces during expansion. Initial patterns on *FLO11* colonies typically appeared as irregular wrinkles developing into a “hub”, a thickening mass of cells in the colony center. After a few days, as the colony expanded beyond the wrinkled hub, and radial wrinkles emerged, some bundled into thicker spoke-like structures. As the colony area increased, radial spokes appeared *de novo* between two existing spokes or by branching (S3 Movie), with apparently quasi-regular spacing (S3 Movie). In contrast, *flo11*Δ colonies appeared smooth, without any obvious surface patterns (Fig. 1B). Wrinkling was also observable on the vertical cross-sections of *FLO11* (but not *flo11*Δ) colonies obtained by cryosectioning (Fig. 3A–F, S7 Figure).

**Figure 4:**
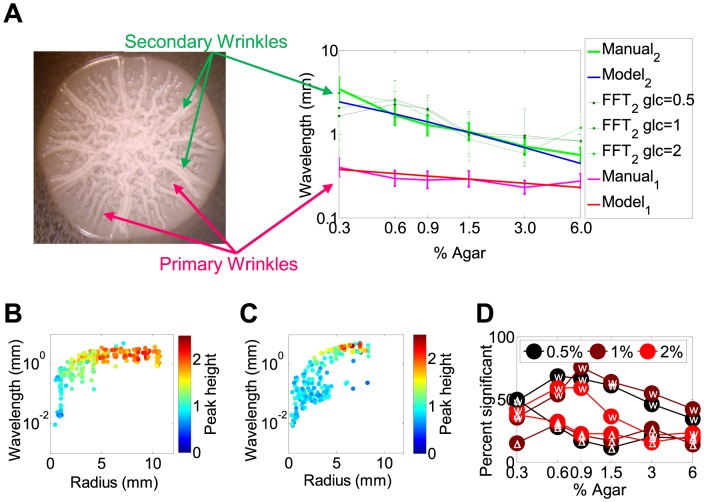
Analysis of the wavelengths of the colony surface patterns. *(A)* The wavelength of spokes (_2_) measured manually and by Fast Fourier Transformation (FFT) decreased with increasing agar density, while the wavelength of primary wrinkles (_1_) from manual measurements were less sensitive to the change of agar density. Since the automated FFT measurement was sensitive to noise in the image, FFT did not detect the small-wavelength primary wrinkles as significant. The wavelength of the wrinkles was shorter than that of the spokes at all agar levels tested. The experimentally measured geometric mean wavelength of spokes matched best the ESVS thick substrate model, while the geometric mean wavelength of primary wrinkles matched the ESVS thin substrate model. The inserted image shows the primary and secondary wrinkles measured at the outer radii, indicated by red and green lines, respectively. *(B)* The Fourier wavelengths plotted as a function of section radius, independent of time for *FLO11* colony at 0.5% glucose and 0.9% agar. *(C)* Same as *(B)*, but for a *flo11Δ* colony at 0.5% glucose and 0.9% agar. Each dot *(C, D)* represented a colony from a replicate and a time point. *(D)* The percent of significant points (Fourier spectral peak height>1.5) plotted as a function of agar. The cell type (*FLO11* or *flo11Δ*) is indicated in white font inside the circles.

To determine whether the distances between wrinkles and spokes were regular, and had a dependence on agar and glucose concentrations, we investigated the surface patterns of individual colonies by manual measurements of inter-wrinkle distances. The narrow distributions of inter-wrinkle and inter-spoke distances suggested regular spacing of these structures (Fig. 4, S8 Figure). Moreover, the number of wrinkles and spokes increased towards the colony edge, indicating that pattern formation tended to preserve inter-wrinkle and inter-spoke arc-lengths rather than arc-angles (S3 Movie). Therefore, the quasi-regular spacing between these structures suggested preference of specific inter-wrinkle and inter-spoke distances. The preferred distance between consecutive spokes decreased with the agar concentration (Fig. 4, S8 Figure).

Next we investigated the mechanisms underlying wrinkle formation. Considering recent evidence for non-uniform cell death underlying bacterial colony wrinkling [17], we tested whether it played a role in the formation of yeast colony surface patterns. We monitored yeast colony expansion on media with SYTOX Green Nucleic Acid Stain, which marks dead cells by green fluorescence [17]. In contrast to bacteria, we observed low levels and uniform distribution of cell death starting from inoculation throughout colony maturation (*see Supporting Methods*), arguing against the role of cell death in pattern formation by yeast colonies (S9 Figure; S4 and S5 Movies). This suggests that an alternative mechanism is responsible for the wrinkling observed in yeast colonies.

A striking observation is the existence of two different spatial scales (i.e., wrinkles and spokes) in the surface patterns of the *FLO11* colonies. This is similar to hierarchical wrinkling in a related mechanical system (Fig. 3G): a thin elastic film on top of a viscoelastic substrate that shrinks [39]. The shrinkage of the substrate relative to the film should cause similar mechanical strain as the stretching of the growing yeast colony (biofilm) relative to the agar substrate. In fact, similar distance-preserving radial wrinkles appear on surfaces where diffusion originating from point-like sources creates instability in a stiff skin [40]. Since the *FLO11* gene mediates surface attachment, *FLO11* cells should be constrained to grow two-dimensionally, forming an elastic skin made of cells and extracellular matrix attached to the viscoelastic agar substrate [41]. In contrast, cells without *FLO11* should expand in three dimensions without any constraints.

Theories of elastic skin-viscoelastic substrate (ESVS) sandwich systems can capture two limiting cases [42]. First, when the substrate is thick relative to the elastic skin then primary wrinkles of wavelength *λ* = *h*(*E_y_*/*E_a_*)^1/3^ should form, where *h* is the thickness of the elastic skin, and *E_y_* and *E_a_* are the Young's Moduli of the skin and the substrate, respectively [39]. Second, if the thicknesses of the skin and substrate are comparable then the wavelength also depends on *H*, the substrate thickness: *λ* = (*hH*)^1/2^(*E_y_*/*E_a_*)^1/6^. If the stress continues to increase, the amplitude of primary wrinkles increases until saturation, after which secondary wrinkles (spokes) start forming according to the same formula, except that the skin thickness is replaced by the primary wrinkles' saturation amplitude. This process of hierarchical wrinkling can continue until wrinkles appear at several length scales [39].

If the theory for hierarchical wrinkling in ESVS sandwich systems applies to *FLO11* colonies, the spatial frequencies should follow formulas as describe above. Indeed, fitting these models indicated that inter-spoke spacing had an inverse dependence on the agar density as expected from the physical ESVS theory of wrinkling when the substrate is thick (Fig. 4A). In contrast, only the thin substrate ESVS model could fit the spacing of primary wrinkles, which was less agar density-dependent (S10 Figure). These findings suggest that primary wrinkle formation involves directly not the agar, but another, thin substrate that sits below the top layer of cells. The density and thereby the elasticity of this substrate could nonetheless correlate with the density of agar, which must enforce the water content and thereby the stiffness of the colony. Indeed, confocal microscopy previously suggested the existence of at least 4 layers (agar, yeast attached to agar, extracellular matrix, and yeast exposed to air) in yeast colonies [41]. Therefore, a likely candidate for the thin substrate is the extracellular matrix squeezed between two cell layers. The spacing of both the spokes and primary wrinkles was independent of glucose concentrations (Fig. 4A, S8 Figure), as expected from the ESVS model.

To further confirm the regularity of patterns on colony surfaces detected by automated image processing, we applied Fast-Fourier Transformations to horizontal sections of the images transformed into polar coordinates (S11 Figure). We noticed that the wavelengths corresponding to dominant frequencies had a tendency to stabilize around specific values towards the outer radii of *FLO11* colonies (Fig. 4B, C). These wavelengths were the most significant and tightly peaked within Fourier spectra for a wide range of agar concentrations (0.6–1.5%) (Fig. 4A, D). Except for the lowest agar concentration (agar = 0.3%), wavelengths obtained for *FLO11* colonies were consistently more significant than for those obtained for *flo11*Δ colonies (Fig. 4D). Finally, the dominant wavelengths (even if sometimes non-significant) tended to decrease with the agar concentration, resembling the agar-dependence of inter-spoke arc-lengths (Fig. 4A), and were only moderately affected by the glucose concentration (Fig. 4A).

### *FLO11* conveys head-to-head competitive advantage during colony expansion

Considering that *FLO11* colonies expanded faster in all sugar-agar combinations than their *flo11*Δ counterparts when grown separately (Fig. 1), we tested whether the two-dimensional constraint on *FLO11* colony growth would also convey a competitive advantage over *flo11*Δ cells during expansion of mixed colonies. In particular, we wanted to know if interactions between these cell types could mitigate the growth advantage of pattern-forming *FLO11* cells.

**Figure 5:**
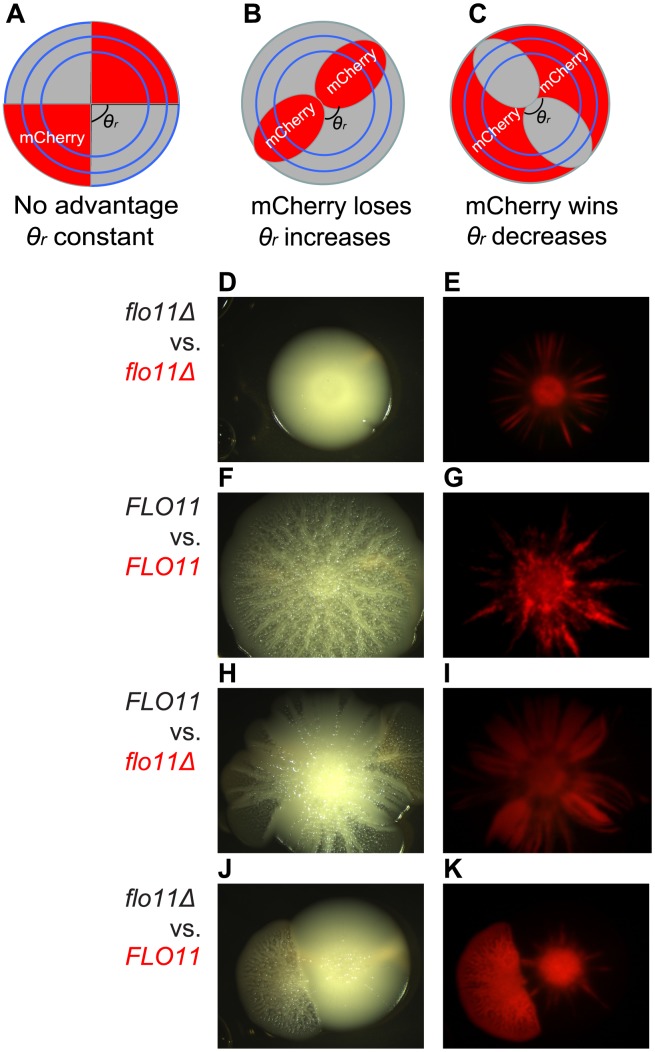
Pattern forming *FLO11 S. cerevisiae* cells out-expand *flo11Δ* cells during head-to-head competition. *(A, B, C)* Schematic showing the range expansion of mixed populations segregated into sectors with constant or gradually changing sector angles along the radius due to fitness differences between the particular sector and the adjacent sectors. *(A)*: Neither population has advantage. *(B)*: Unlabeled population has advantage. *(C)*: Red-labeled population has advantage. *(D, E)* A small competitive advantage of unlabeled cells is observed between isogenic cells of unlabeled and mCherry-labeled *flo11Δ* cells. *(F, G)* A small competitive advantage of unlabeled cells is observed between unlabeled and mCherry labeled *FLO11* cells. *(H, I)* Unlabeled *FLO11* cells out-expanded mCherry-labeled *flo11Δ* cells with a conspicuous increase in the unlabeled sector angle, in comparison to minimal competition between isogenic cells (see below). *(J, K)* Reverse labeling of *(H, I)* showed that mCherry-labeled *FLO11* cells overtook the mixed colonies after some time, despite of the initial lack of expansion advantage against unlabeled *flo11Δ* cells. Bright field *(D, F, H, J)* and mCherry *(E, G, I, K)* were shown respectively. Contrast is adjusted in Adobe Photoshop CS for mCherry images. All cells were grown on 1.0% agar, 0.5% galactose YPGal plates.

Previously, Korolev and colleagues [43] showed that two differently labeled *S. cerevisiae* cell types should segregate into single-colored sectors as initial spatial non-homogeneities amplify through a “founder effect” during colony expansion. The boundaries of such single-colored sectors should reveal any competitive advantage between the two cell types (or lack thereof). Specifically, straight sector boundaries indicate that sectors occupy approximately the same arc-angle *θ*(*r*) around the colony's periphery over time (Fig. 5A), meaning that neither cell type has competitive advantage over the other. However, if the sector boundaries occupied by unlabeled cells curve outwards then their arc-angle *θ*(*r*) increases at the expense of the arc-angle occupied by the mCherry-labeled cells, meaning that the unlabeled cells have a competitive advantage (Fig. 5B). The opposite is true if the unlabeled sector boundaries curve inward (Fig. 5C).

We used these theoretical results to judge whether pattern formation in our experimental system was associated with an advantage during head-to-head competition. To examine competition in mixed colonies, we labeled either *FLO11* cells or *flo11*Δ cells, placing a chromosomally integrated mCherry reporter under the control of an extra copy of the *GAL1* promoter into each cell type (*see* *Methods*).

We found that well-mixed liquid cultures (1∶1 ratio) of red-labeled and unlabeled cells inoculated onto agar plates resulted in distinct red and unlabeled sectors (Fig. 5). Examining the sectors that formed when mCherry-labeled *FLO11* cells were mixed with unlabeled *FLO11* cells (or when mCherry-labeled and unlabeled *flo11*Δ cells were mixed), indicated minimal competitive advantage, except for a small fitness cost associated with mCherry expression (Fig. 5D–G). Importantly, the mixtures of labeled and unlabeled *FLO11* cells preserved the colony expansion and pattern formation characteristics of *FLO11* cells grown alone under the same conditions (Fig. 5, S12 Figure).

When a mixture of *flo11*Δ (labeled) and *FLO11* (unlabeled) cells were inoculated onto a plate, *FLO11* sectors not only preserved the surface patterning (visualized in bright field), but also displayed a strong outward curvature at the expense of *flo11*Δ sectors (Fig. 5H, I). The arc-angle θ of the *flo11*Δ colony (mCherry labeled) decreased more with the radius (Fig. 5I) compared to the control mixtures (Fig. 5D–G). Interestingly, however, mCherry-labeled *FLO11* cells seemed to gain competitive advantage against *flo11*Δ cells only after colonies grew for a substantial time, indicating that a sufficiently large sector of these cells had to be established, or the sugar levels on the plate had to drop before they could outcompete *flo11*Δ cells. Once that happened, not only did the *FLO11* gene convey a competitive advantage, but the *FLO11* cells eventually enveloped the *flo11*Δ sector (visualized by mCherry in Fig. 5I) by coalescing with the adjacent *FLO11* sector (Fig. 5H). This halted the spread of the *flo11*Δ colony (Figure 5I). We observed similar behavior with reverse labeling (Fig. 5J, K) and in repeat experiments (S13, S14 Figures and S6 Movie), once again confirming that *FLO11* cells gain advantage after an initial delay. Varying the percentage of *flo11*Δ versus *FLO11* cells did not alter these results, as long as pattern formation appeared in the *FLO11* sector. These data, taken together with the similarity of *FLO11* and *flo11*Δ cell sizes and growth rates in liquid cultures (Supporting S4 Figure), and microcolony expansion rates (S1 and S2 Movies), indicate that the two-dimensionally constrained expansion mode of *FLO11* cells gives them advantage over *flo11*Δ cells when they form mixed colonies on agar plates.

Overall, we found that *FLO11*-enforced two-dimensional colony expansion conveyed head-to-head competitive advantage against *flo11*Δ cells. The widening of *FLO11* sectors and the envelopment of the outside rim of *flo11*Δ cells by *FLO11* cells indicate that *FLO11* cells robustly out-competed *flo11*Δ cells during colony expansion.

## Discussion

Microbial pattern formation has received considerable attention over the last century and even before [44], [45]. In the 1990s there was a surge of interest in quantitative characterization of microbial colony patterns as physicists forged connections to non-equilibrium growth phenomena [46], [47]. Recently, interest in microbial patterns has reemerged again, as advanced genetic and imaging methods permitted screening of mutations responsible for macroscopic pattern formation while observing single cells in developing colonies [17], [41], [48]. Although many recent studies describe phenomenological associations of various molecular and physical processes with pattern formation, it is unclear if a unifying theme underlies these observations. We propose here that constraining the dimensionality of colony expansion could be such a unifying theme.

Deeper understanding of colony development requires automated image processing methods to extract quantitative data from images [17], [32] and develop mathematical or physical models to establish connections with related processes in physical systems. This work represents a step in this direction by systematically extracting and quantifying features in expanding yeast colonies.

We studied, by quantitative methods, three salient features of yeast colonies during their expansion on agar plates: colony size, rim irregularity and the pattern emerging on the colony surface. By comparing *FLO11* cells to otherwise isogenic *flo11*Δ cells, we observed that the *FLO11* gene increases the rate of colony expansion, enhances rim irregularity, and is required for pattern formation in all the sugar and agar concentrations tested. These trends were consistent among three different trials with glucose (Fig. 1), as well as a trial with galactose, indicating the robustness of colony expansion features (S3 Figure). Seeking a unifying explanation for these experimental observations, we proposed a simple mathematical model to capture these differences. Our model qualitatively reproduces colony expansion curves under different growth conditions. In addition, it suggests a possible explanation for the formation of petals on the *FLO11* colony boundary, which can occur due to the competition among cells for common nutrients. Random advantage of some cells over others can result from any fluctuating factor (spatial inhomogeneities in cell density, nutrients or agar concentration), which is then amplified as petals grow, consuming and depleting nutrients from their vicinity. Depletion of nutrients between petals prevents the growth of cells, and leads to branch-like structures, somewhat similar to what has been observed in bacterial colonies [31], [33], [49].

Moreover, we propose that the patterns observed on the colony surface arise by hierarchical wrinkling similar to sandwich systems consisting of an elastic skin on top of a viscoelastic substrate. Microbial biofilms typically contain an extracellular matrix that cells secrete, creating a connective medium across the colony that acts as an elastic skin [50]. The expansion of the colony attached to the agar can cause strain, which generates hierarchical wrinkling. However, the yeast-colony-agar system is more complex than bilayer ESVS systems that current theories can capture. Therefore, investigating patterns on microbial colony surfaces could inspire the development of new physical theories of wrinkling in multi-layered systems.

Overall, we propose the 2D agar-attached mode of colony expansion as the common underlying cause of the observed phenomena (faster colony expansion, rim irregularity, and pattern formation) in *FLO11* cells. In addition, we find that two-dimensional expansion provides competitive advantage during head-to-head competition with *flo11*Δ colonies that expand in an unconstrained manner. Indeed, the unifying theme of two-dimensional expansion is intuitive for at least three reasons. First, for the same number of cells to fit in a 2D sheet compared to a 3D hump, the shape must expand over a wider area. Second, 2D expansion with agar attachment should be sensitive to local impurities and form petals, while 3D expansion can “overpass” such roadblocks in the 3^rd^ (vertical) dimension. Finally, 2D agar-attached cell expansion creates a system equivalent to an elastic film spread on top of a shrinking viscoelastic substrate, causing hierarchical wrinkling, which is not true for unconstrained 3D expansion.

Biofilm wrinkling in other organisms has been shown to perform specific biological functions. For example, wrinkling could be induced under anoxic environment, increasing the oxygenation in *Pseudomonas aeruginosa* colonies [51]. The channels underneath wrinkles were suggested to transport liquid within *Bacillus subtilis* biofilms [52]. Moreover, enforcing two-dimensional expansion may reduce the evolutionary conflict among cells as they compete for resources located below (nutrients) and above the colony (oxygen) [11], [12]. Yet, identifying the function of biofilm features necessitates knowing the mechanisms underlying their formation, such as we propose for yeast colonies. Without a physical sciences perspective, molecular and cell biological considerations will be insufficient to understand the intricacies of biofilm formation. Overall, this work is an example for how the restriction of dimensionality during expansion can create complex patterns and provide a competitive advantage that would not exist for dimensionally unconstrained growth.

## Methods

### Cells, media and growth conditions

Haploid *S. cerevisiae* TBR1 (Σ1278b, matα, *FLO11*, tryp) and TBR5 (Σ1278b, matα, *flo11*Δ, tryp) cells were used as parental strains. 0.5 µl of *FLO11* or *flo11*Δ (OD≈0.3) cells was inoculated onto plates (Fisher scientific, Cat#: 0875714G) containing ∼15 ml Yeast Extract Peptone Dextrose (YPD) substrate with combinations of (1.5%, 3.0% or 6.0%) agar and glucose (0.5%, 1.0% or 2.0%) were used for colony expansion. The same procedure was repeated for preparing Yeast Extract Peptone Galactose (YPGal) plates, except using galactose instead of glucose. YPD 6-well plates (BD Falcon, Cat#: 353046) were prepared with substrate containing various agar and glucose concentrations for cryosectioning and manual measurement and FFT analysis on the wavelengths of wrinkles and spokes. For competition experiments, 0.5 µl mixed culture at 1∶1 ratio (OD≈0.3) of *FLO11* and *flo11*Δ (one of which was labeled by mCherry) was inoculated in the center of YPGal plates with 1.0% agar and 0.5% galactose. Plates were incubated at 30°C (Barnstead Lab-Line stationary incubator).

### Fluorescently labeled cells

We integrated mCherry into the native GAL1 locus of *FLO11* or *flo11*Δ cells, respectively, using the histidine auxotroph marker. Transformation was performed with a modified lithium acetate procedure [53]. Synthetic drop-out media (SD-his-tryp) (all reagents from Sigma, Inc.) was used for selection. Cells were incubated under 30°C, shaking at 300 rpm (311DS LabNet shaking Incubator).

### Plate imaging, microscopy

Plates of parental strains or mCherry-labeled cells were imaged under a BioRad imager or Leica MZ6 stereo microscope. Cryosectioned slices were imaged under a Nikon Eclipse TE2000-E microscope and montaged in Adobe Photoshop CS.

### Image processing

To analyze colony images, we detected the plate in each image by thresholding the image and searching for a circular object. This was done by defining a cost for deviation from circularity, and choosing the threshold that minimized it. We then removed the plate from the image and detected the colony by binarizing the image across a threshold value. This value was determined from the image intensity histogram by separating the peaks corresponding to image background and foreground.

### Analysis on colony expansion time course and the non-circularity of colony rim

To determine colony irregularities, we applied two different methods. Both methods are scale-invariant and increase as irregularities at the boundary increase, relying on the deviations of the shape from a circle. First, we defined P2A as the inverse of “isoperimetric quotient”:
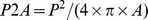
where *P* is the perimeter of the colony calculated by counting 8-connected pixels on colony boundary, and *A* is the area of the colony calculated by counting the total number of pixels in the colony. Second, we defined Boundary Fluctuation (BF) by plotting the distance of each point on the colony boundary from the centroid (*r*) as a 2-D curve in polar coordinates, versus the polar angle θ. The BF was then defined as the coefficient of variation of the *r*(θ) curve, *BF = std*(*r*)*/mean*(*r*), over all values of θ.

### Computer simulations of colony expansion

We propose a model based on models of colony formation in bacteria (Palumbo et al., 1971). Cells are treated as a medium that can diffuse via a nonlinear diffusion constant, while consuming glucose to grow. Glucose can diffuse with a constant rate. The equations of the system are:
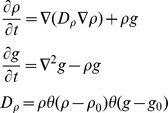
where *ρ* is cell density, *g* is glucose concentration, 18 is the rate of cell growth and nutrient consumption. The cellular diffusion constant 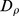 involves the Heaviside step function *θ(x)*, which is 1 for positive *x*, and 0 otherwise. Simulations were done using Euler method (*dt = 0.064, Δx = 0.8*) with Neumann boundary conditions in a 160×160 square lattice. Initially glucose concentration was constant and cell density was set to zero except inside a circle of radius 16, where density was chosen at random from a flat distribution in the range 0.03<*ρ*<0.09. The remaining model parameters are *ρ*_0_ = 0.01, *g*_0_ = 0.014.

### Colony freezing and cryosectioning

Colony blocks were immersed in clear frozen section compound (VWR, CA95057-83B), frozen in a HistoChill Cryobath (SP Scientific, FTS system), and then sectionedat4 µm thickness.

### Physical models for *S. cerevisae* colony wrinkling

We applied the ESVS thin and think substrate models [39], [40], [42], [54], estimating the agar Young Modulus based on the relationship [55]

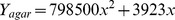
between Young's Modulus and the density of agar, *X*. Young's modulus for several agar densities was estimated by fitting a quadratic function to previous nanoindentation-based measurements [55] considering that Young's modulus for the viscoelastic agar substrate is the magnitude of the complex Young's modulus calculated from:
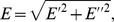
where E′ is the storage modulus and E″ is the loss modulus [55].

Based on these considerations, we used the following relationship for fitting the ESVS thick substrate model:
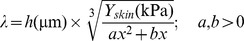
where *x* indicates the percentage of agar, *h* is the thickness of the skin and *λ* is the wrinkle wavelength for *S. cerevisiae*.

For fitting the ESVS thin model, we used the following formula:
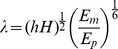
where *H* is the thickness of the substrate.

### Manual measurement on the wavelength of colony patterns

The wavelengths of the wrinkles and spokes were obtained from
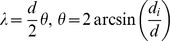
where *d* is the diameter of the circle that harbored the measured arc-length distances *d_i_*. θ and λ are angles and wavelengths for wrinkles and spokes. Total number of arc-length distances of wrinkles or spokes measured were from 50 to 200 for each agar condition. The distances (*d_i_*) between adjacent wrinkles or adjacent spokes were measured around a circle near the colony rim. The diameter of the circle (*d*) and the diameter of the plate in the image were also measured. Knowing the actual diameter of the plate (3.5 cm), we could therefore estimate the actual radius of the circle (*d*), as well as the inter-spoke and inter-wrinkle distances (*d_i_*).

### Fast Fourier Transformation analysis

Images were converted to grayscale and colonies were segmented based on pixel brightness and radius, using a Gaussian mixture model. Yeast colonies were converted to radial coordinates using the *imgpolarcoord* Matlab file written by Juan Carlos Gutierrez and Javier Montoya. The image data lines at each radius within a section were Fast Fourier Transformed (FFT). The mean and standard deviations of the FFT intensities within each section were then calculated, and used to create the mean spectra and the standard deviation of the spectra. Finally, the heights of the peaks were calculated as 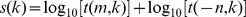, where *t* represents a *t*-statistic defined as:
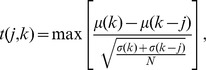
where 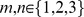. The mean and standard deviation of spectral intensity corresponding to *k* are 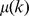, 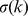, while *N* is the number of spectra used to calculate 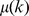 and 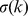. The peak with highest *s* was chosen as the frequency of spokes. Wavelengths were then calculated by dividing the outermost perimeter of a section by the number of spokes (or oscillations) corresponding to the frequency.

## Supporting Information

S1 Figure **Maximal colony area, colony irregularity and their times of saturation as functions of agar and glucose concentration.** *(A–D)* The maximum colony area *(A, B)* and the irregularity of the colony rim *(C, D)* of both *FLO11 (A, C)* and *flo11Δ (B, D)* colonies inversely depended on agar density. The maximum colony area *(A, B)* increased and the maximum irregularity of the colony rim *(C, D)* decreased with glucose concentration, regardless of *FLO11*. *(E–H)* Colonies approached the maximum area *(E, F)* and the irregularity *(G, H)* faster at lower agar density regardless of *FLO11*.
(PDF)

S2 Figure **Derivative of the colony area versus time indicated the change in convexity.** *(A–C)* Time derivative of the experimentally measured *FLO11* colony area at agar concentration = 1.5% indicated glucose-dependent increase of colony expansion curve convexity *(* *Fig. 1C* *)*. Convexity is indicated by the presence of a peak at higher *(S13B, C Figure)* and lack thereof at lower *(S13A Figure)* glucose concentrations, respectively. *(D–F)* Time derivative of colony area from simulations in *Figure 2* also indicated an overall sugar-dependent increase in convexity. This is indicated by the appearance of a peak at higher glucose levels *(S13E, F Figure)*.
(PDF)

S3 Figure ***FLO11*** **colonies expanded faster and were more irregular than** ***flo11Δ*** **colonies on YPGal plates.** *(A–F)* The colony size *(A)* and the irregularity of *FLO11* colony measured by P2A *(C)* and BF methods *(E)* increased along the time course, with higher value compared to *flo11Δ (B, D, F)* at most conditions. The conditions included three agar densities at 1.5%, 3.0%, 6.0% (indicated as A15, A30 and A60 respectively in the figure legend) and three glucose concentrations at 0.5%, 1.0%, and 2.0% (indicated as G05, G10, and G20 respectively in the figure legend).
(PDF)

S4 Figure **No significant growth difference between** ***FLO11*** **and** ***flo11Δ S. cerevisiae*** **cells in liquid media.** On 0.5% galactose YPGal liquid media, *(A)* No significant difference throughout the 57-hour growth curves between *FLO11* and *flo11Δ* cells. Three independent replicates of *FLO11* (red) and *flo11Δ* (green) were tested to ontain error bars for the average curve. *(B, C, D, E)* The distributions of cells and clumps diameters between *FLO11 (B, C)* and *flo11Δ (D, E)* at 28 hours *(B, D)* in exponential growth and at 48 hours *(C, E)* in stationary phase were similar.
(PDF)

S5 Figure **Colony irregularity measured by Boundary Fluctuation is higher for** ***FLO11*** **cells compared to** ***flo11Δ*** **cells.** *(A)* Images of *FLO11* and *flo11Δ* colonies were segmented and the boundaries were quantitatively analyzed in polar coordinates. *(B)* The boundary fluctuation of *FLO11* (red curves) was inversely increasing with both agar and glucose concentrations. Minimal fluctuation was observed at the colony boundaries of *flo11Δ* mutants (green curves), compared to the conspicuous fluctuation at the *FLO11* boundaries (red curves). Thinner curves were different replicates and thicker curves were their average up to a time when all the replicates were present.
(PDF)

S6 Figure **Mathematical model of** ***flo11Δ*** **colony expansion.** *(A–C)* Snapshots of colonies from simulations with a phenomenological third dimension (modeled by inclusion of a cell density-dependent escape term). The images show cell density at the agar surface at the end of a simulation for three different initial glucose levels. Introducing the escape term caused smaller colony size compared to simulations of *FLO11* colonies without the escape term *(* *Fig. 2* *)*. *(D–F)* Time-courses of colony area (scaled to simulation box) for the three glucose conditions indicated. The maximum and overall area was higher for higher glucose concentrations, in agreement with the experimental results in Fig. 1. *(G–I)* Time-courses of P2A for the three glucose conditions.
(PDF)

S7 Figure **The cross-sectional view of the hierarchical wrinkles of** ***FLO11*** **colony expanding at various agar concentrations.** *(A, F, K) FLO11* colonies on 0.6%, 1.5%, or 3.0% YPD agar plates were cryosectioned at across-spokes (blue indicated estimated location) and radial (yellow indicated estimated location) orientations. Scale bars were 7.5 mm. *(B, D, G, I, L, N)* On 0.6%, 1.5%, or 3.0% agar, the cross-sectional view of the across-spokes (blue) oriented section from *FLO11* colonies, two degrees of wrinkling composed of shorter-wavelength wrinkles at the surface of the colony (red box) and the longer-wavelength spokes underneath it (green box). *(C, E, H, J, M, O)* On 0.6%, 1.5%, or 3.0% YPD agar plates, only shorter-wavelength wrinkles were shown on the radial (yellow) cross-sectional view of the *FLO11* colonies. *(B, C, G, H, L, M)* Images were taken under Nikon Eclipse TE2000-E Microscope, *(D, E, I, J, N, O)* Images were taken under Leica MZ6 stereo microscope *(See Methods)*. Overall, the hierarchical wrinkles were observed on *FLO11* colonies on various agar conditions. *(B–E, G–J, L–O)* Scale bar were 500 µm.
(PDF)

S8 Figure **The distribution of inter-spoke distances (wavelengths) of** ***FLO11*** **colony depended inversely on agar density.** *(A, B)* The wavelength distribution for both primary wrinkles (1) *(A)* and secondary wrinkles (spokes) (2) *(B)* were insensitive to the change of glucose concentrations. *(C, D)* The wavelength distribution for secondary wrinkles (spokes) (2) *(D)* shifted to shorter mean wavelengths with the increase in agar density. The wavelength distribution of the primary wrinkles (1) *(C)* was insensitive to the change of agar density. Primary and secondary wrinkles (spokes) were indicated by (1) and (2), respectively, in the figure legend. The unit for the wavelength in all panels was µm.
(PDF)

S9 Figure **Cell death was minimal and uniform during** ***FLO11*** **colony expansion.** *(A)* No particular death pattern has been detected on 0.6% agar and 1.0% glucose YPD plate with 5 µM Sytox green nucleic acid, at 19:40 hours, 26:20 hours, 30:50 hours, 41:40 hours and 67:40 hours after inoculation (images extracted from S4 and S5 Movies). *(B–C) FLO11* colony incubated with 5 µM Sytox was imaged before (at 14 hours after inoculation) *(B)* and after adding of 3% H_2_O_2_ (at 15 hours after inoculation) *(C)* as positive control. Minimal cell death was detected (bottom panel) in the former, and uniform cell death was observed for the latter at 36 hours. *(D–E)* In the absence of Sytox as a negative control, no fluorescence was observed before (at 20 hours) *(D)* or after (at 42 hours) *(E)* adding 3% H_2_O_2_ at 21 hours. *(F)* No fluorescence was observed for *FLO11* colony with neither Sytox nor H_2_O_2_. *(A–F)* Bright field and FITC channel were shown on the top and bottom panels respectively.
(PDF)

S10 Figure **ESVS thick and thin substrate model fits to experimentally measured wavelengths for primary and secondary wrinkles.** The geometric mean wavelength of the primary *(A, C, E)* and secondary wrinkles *(B, D, F)* was plotted in magenta and green, respectively for FLO11 colonies growing on YPD media with various agar densities (0.3%, 0.6%, 0.9%, 1.5%, 3.0%, and 6.0%). *(A, B)* ESVS thick substrate model fits to primary *(A)* and secondary wrinkles *(B)* with R-square values of −0.23 and 0.89, respectively. *(C, D)* ESVS thin substrate model fit to primary *(C)* and secondary wrinkles *(D)* with R-square values of 0.63 and 0.6 respectively. *(E, F)* The agar-independent model fit both primary *(E)* and secondary wrinkles *(F)* with R-square values of 0. We obtained very similar results when using the arithmetic mean.
(PDF)

S11 Figure **Pipeline of FFT analysis for** ***FLO11*** **and** ***flo11Δ*** **colonies.** *(A)* Sample images of *FLO11* (top) and *flo11Δ* (bottom) colonies used for analysis. *(B)* Original images were transformed into polar coordinates, and divided into 10 horizontal sections. *(C)* The mean spectra and the standard deviation of the spectra resulting from Fast Fourier Transformation analysis within each section. *(D)* Peak heights within the spectra were calculated based on t-test scores and the highest peaks were kept as the secondary and primary frequencies. Large circles marked the secondary frequencies, while smaller circles marked the primary frequencies. The top or bottom row corresponded to a *FLO11* or *flo11Δ* colony. Both colonies were grown on 0.5% glucose, 0.9% agar YPD plates at 4 days. *(E)* FFT analysis processed images of *FLO11* (top) and *flo11Δ* (bottom) colonies.
(PDF)

S12 Figure **The controls for competition between pattern forming** ***FLO11 S. cerevisiae*** **colonies and** ***flo11Δ*** **during head-to-head competition.** *(A)* On 1.0% Agar YPGal plates with 0.5% galactose, unlabeled *FLO11* expanded with regular pattern formation. *(B)* Pattern forming mCherry labeled *FLO11* colony was visualized by mCherry fluorescence throughout the colony (right panel). *(C)* Unlabeled *flo11Δ* colony expanded without pattern. *(D)* mCherry labeled *flo11Δ* colony was visualized by mCherry fluorescence (right panel) with no pattern formation. Contrast is adjusted in photoshop for mCherry images.
(PDF)

S13 Figure ***FLO11 S. cerevisiae*** **cells out-expand** ***flo11Δ*** **cells during head-to-head competition.** Replicate #2 of competing *FLO11* and *flo11Δ* cells on 1.0% agar, 0.5% galactose YPGal plates. *(A, B)* Minimal competition between isogenic strains of unlabeled and mCherry labeled *flo11Δ* sectors. *(C, D)* Minimal competition was observed between unlabeled and mCherry labeled *FLO11* sectors. *(E, F)* unlabeled *FLO11* sector out-expanded mCherry labeled *flo11Δ* sectors with a conspicuous increase in the unlabeled sector angle. *(G, H)* Reverse labeling of *(E, F)*. Bright field *(A, C, E, G)* and mCherry *(B, D, F, H)* were shown respectively. Contrast is adjusted in Adobe Photoshop CS for mCherry images.
(PDF)

S14 Figure ***FLO11 S. cerevisiae*** **cells out-expand** ***flo11Δ*** **cells during head-to-head competition.** Replicate #3 of competing *FLO11* and *flo11Δ* cells on 1.0% agar, 0.5% galactose YPGal plates. *(A, B)* Minimal competition between isogenic strains of unlabeled and mCherry labeled *flo11Δ* sectors. *(C, D)* Minimal competition was observed between unlabeled and mCherry labeled *FLO11* sectors. *(E, F)* unlabeled *FLO11* sector out-expanded mCherry labeled *flo11Δ* sectors with a conspicuous increase in the unlabeled sector angle. *(G, H)* Reverse labeling of *(E, F)*. Bright field *(A, C, E, G)* and mCherry *(B, D, F, H)* were shown respectively. Contrast is adjusted in Adobe Photoshop CS for mCherry images.
(PDF)

S1 Movie **Movies of early colony expansion for** ***FLO11*** **cells.** Early colony expansion of *FLO11* cells immediately after placing cells onto 0.5% galactose and 1.0% agar plates from OD_600_ = 0.001 liquid culture of equal composition, except without agar.
(AVI)

S2 Movie **Movies of early colony expansion for** ***flo11Δ*** **cells.** Early colony expansion of *flo11Δ* cells immediately after placing cells onto 0.5% galactose and 1.0% agar plates from OD_600_ = 0.001 liquid culture of equal composition, except without agar.
(AVI)

S3 Movie **Movie of pattern formation for** ***FLO11*** **colony.** *FLO11* cells gradually developed spokes extending from the center to the rim of the colony and maintained certain dominant frequencies throughout time via branching from original spokes or emergence of new ones.
(MP4)

S4 Movie **Movie of early** ***FLO11*** **colony expansion.** Cells expanding into a colony on 0.6% agar and 1.0% glucose YPD media imaged in bright field (BF).
(WMV)

S5 Movie **Movie of cell death (FITC) during the early phase of** ***FLO11*** **colony expansion.** The same cells as in S3 Movie, labeled with 5 µM Sytox green nucleic acid dye (FITC channel). We observed minimal and uniformly distributed cell death over the first 72 hours, without any distinct patterns (FITC channel), even though wrinkle formation had begun already.
(WMV)

S6 Movie **Movie of competition between pattern forming** ***FLO11*** **and** ***flo11Δ*** **cells.** Unlabeled *FLO11* cells out-competed mCherry-labeled *flo11Δ* cells in mixture during the first 30 hours of competitive expansion on 0.6% Agar, 1.0% galactose YPGal media.
(WMV)

S1 Dataset This compressed file contains tab-delimited text data files and other documents in 5 subfolders: (i) Colony_Area&Irregularity contains colony area and irregularity values for various glucose and agar combinations (indicated by the file names, for example A15G05TBR1 means *FLO11* colony in 1.5% agar and 0.5% glucose); (ii) PDEmodel_python contains the files for numerically solving the PDE model of colony expansion; (iii) WrinkleDistance_Detected contains tab-delimited data of colony surface patterns detected using FFT; (iv) WrinkleDistance_Measured contains tab-delimited data of inter-wrinkle distances (human measurement); (v) FFT contains an example for image segmentation followed by Fast Fourier Transformation to extract significant frequencies/wavelengths.
(ZIP)

S1 Text **Supporting materials and methods.** This document contains additional methods describing the procedures for recording the Supporting Movies.
(PDF)
