# Supplementary figures and images for: Two-Dimensionality of Yeast Colony Expansion Accompanied by Pattern Formation
Source: PLoS Comput Biol. 2014 Dec 11;10(12):e1003979. doi: 10.1371/journal.pcbi.1003979 (PMC4263361; doi:10.1371/journal.pcbi.1003979)

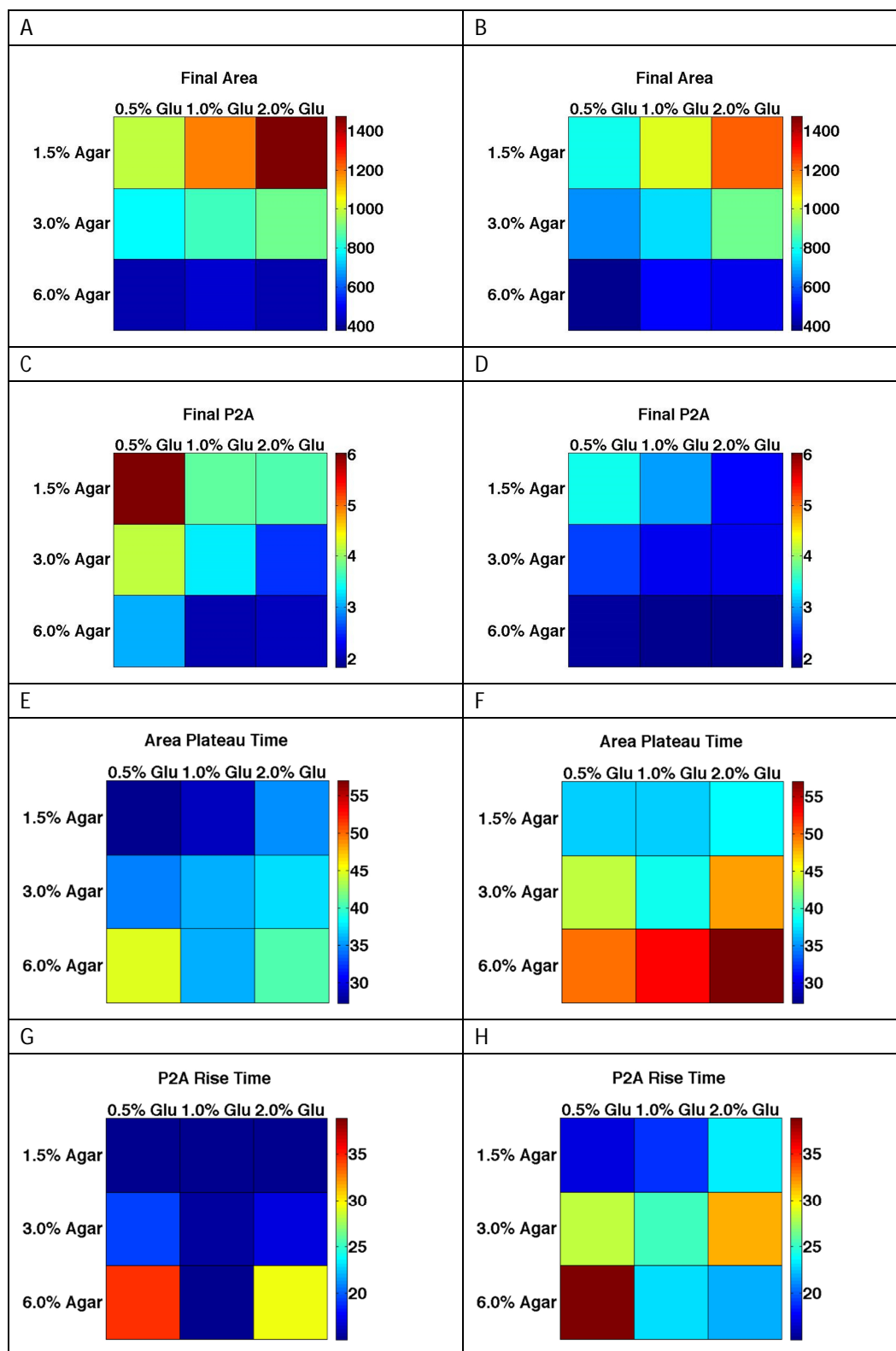

**Supporting Figure S1.**

Supplement: S1 Figure — Maximal colony area, colony irregularity and their times of saturation as functions of agar and glucose concentration. (A–D) The maximum colony area (A, B) and the irregularity of the colony rim (C, D) of both FLO11 (A, C) and flo11Δ (B, D) colonies inversely depended on agar density. The maximum colony area (A, B) increased and the maximum irregularity of the colony rim (C, D) decreased with glucose concentration, regardless of FLO11. (E–H) Colonies approached the maximum area (E, F) and the irregularity (G, H) faster at lower agar density regardless of FLO11. (PDF) [file pcbi.1003979.s001.pdf]

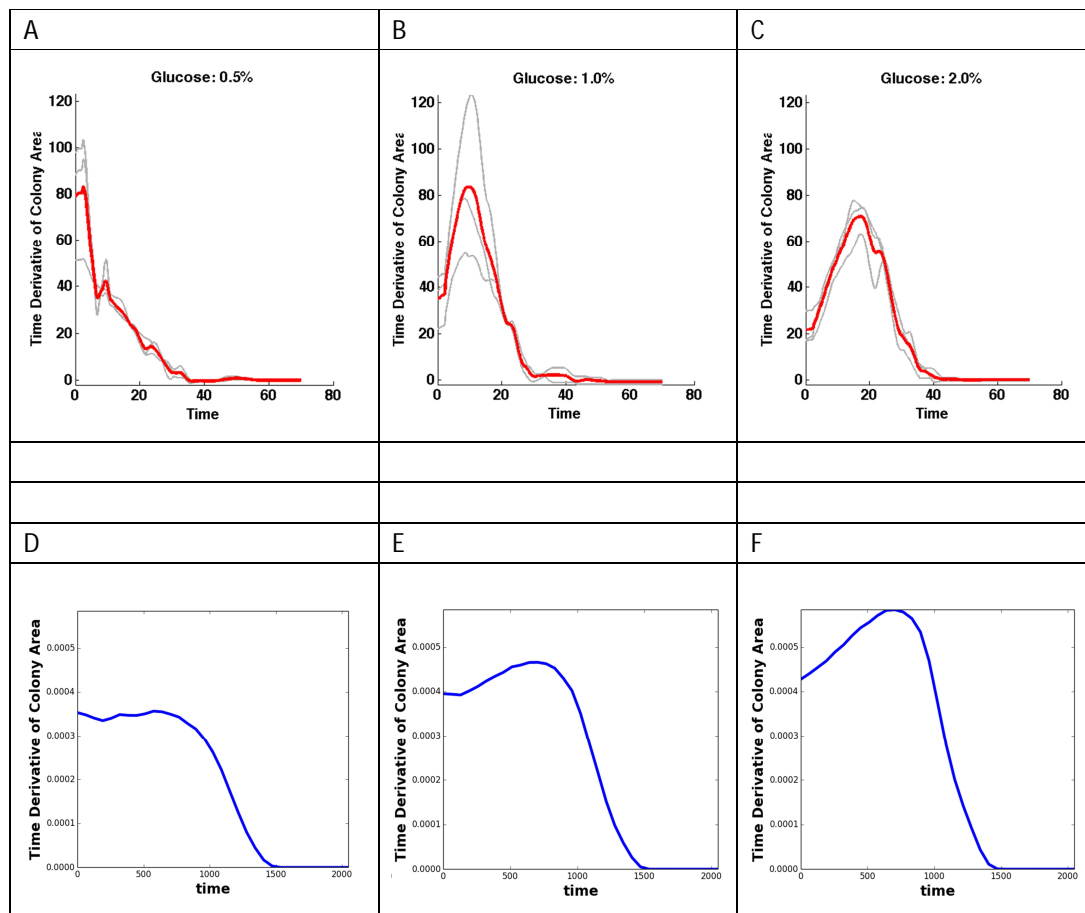

**Supporting Figure S2.**

Supplement: S2 Figure — Derivative of the colony area versus time indicated the change in convexity. (A–C) Time derivative of the experimentally measured FLO11 colony area at agar concentration = 1.5% indicated glucose-dependent increase of colony expansion curve convexity ( Fig. 1C ). Convexity is indicated by the presence of a peak at higher (S13B, C Figure) and lack thereof at lower (S13A Figure) glucose concentrations, respectively. (D–F) Time derivative of colony area from simulations in Figure 2 also indicated an overall sugar-dependent increase in convexity. This is indicated by the appearance of a peak at higher glucose levels (S13E, F Figure). (PDF) [file pcbi.1003979.s002.pdf]

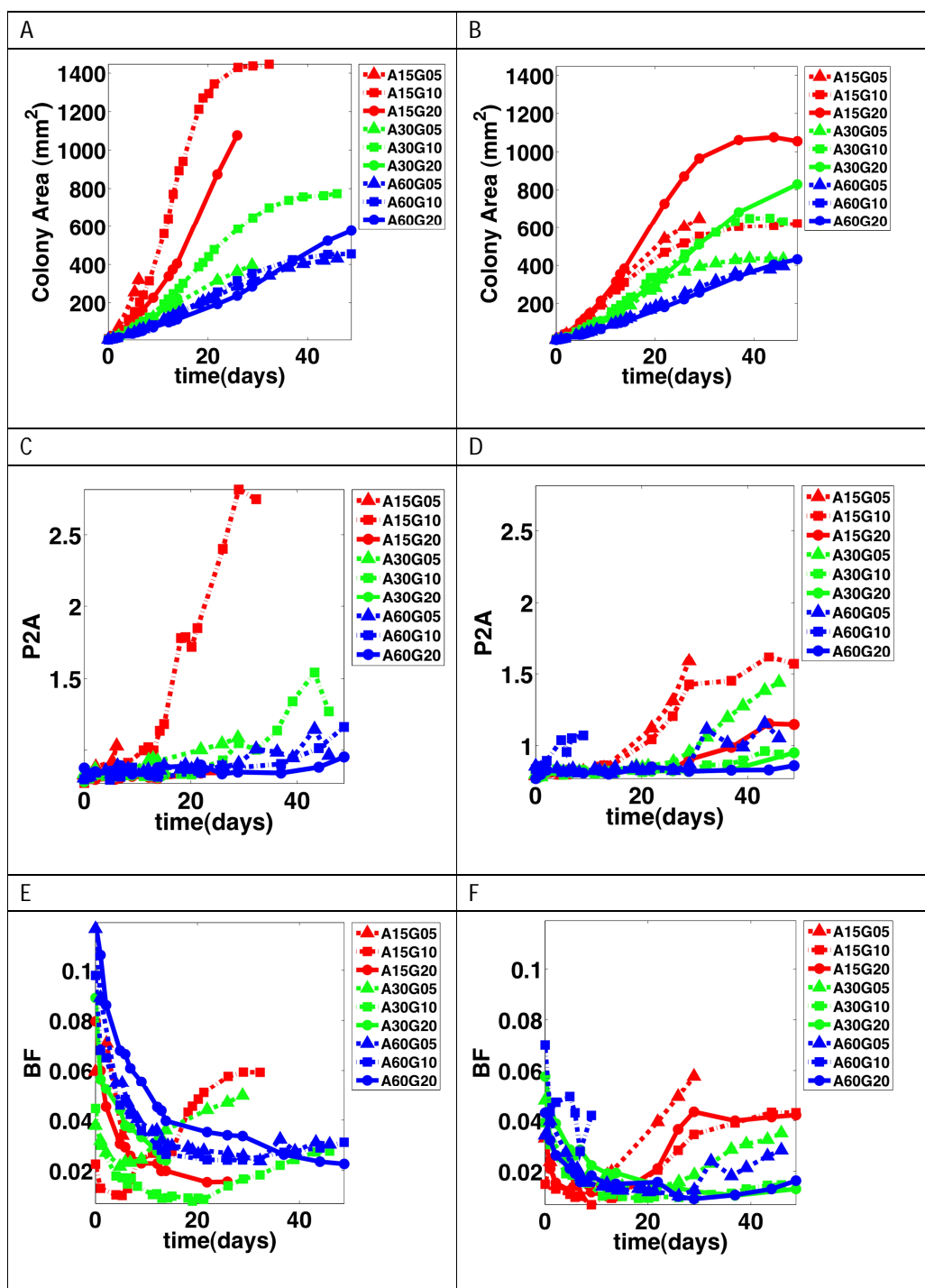

Supporting Figure S3.

Supplement: S3 Figure — FLO11 colonies expanded faster and were more irregular than flo11Δ colonies on YPGal plates. (A–F) The colony size (A) and the irregularity of FLO11 colony measured by P2A (C) and BF methods (E) increased along the time course, with higher value compared to flo11Δ (B, D, F) at most conditions. The conditions included three agar densities at 1.5%, 3.0%, 6.0% (indicated as A15, A30 and A60 respectively in the figure legend) and three glucose concentrations at 0.5%, 1.0%, and 2.0% (indicated as G05, G10, and G20 respectively in the figure legend). (PDF) [file pcbi.1003979.s003.pdf]

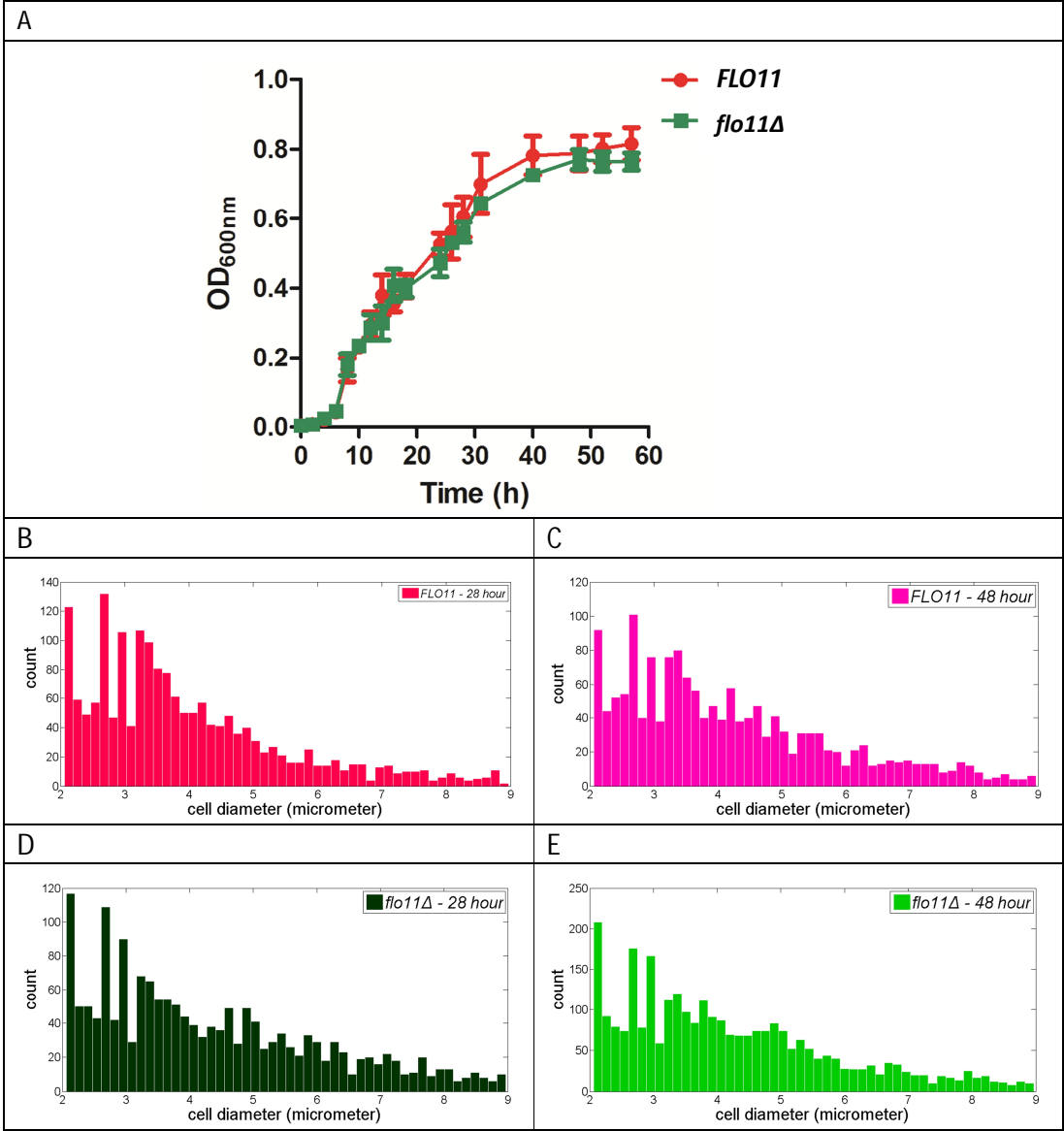

**Supporting Figure S4.**

Supplement: S4 Figure — No significant growth difference between FLO11 and flo11Δ S. cerevisiae cells in liquid media. On 0.5% galactose YPGal liquid media, (A) No significant difference throughout the 57-hour growth curves between FLO11 and flo11Δ cells. Three independent replicates of FLO11 (red) and flo11Δ (green) were tested to ontain error bars for the average curve. (B, C, D, E) The distributions of cells and clumps diameters between FLO11 (B, C) and flo11Δ (D, E) at 28 hours (B, D) in exponential growth and at 48 hours (C, E) in stationary phase were similar. (PDF) [file pcbi.1003979.s004.pdf]

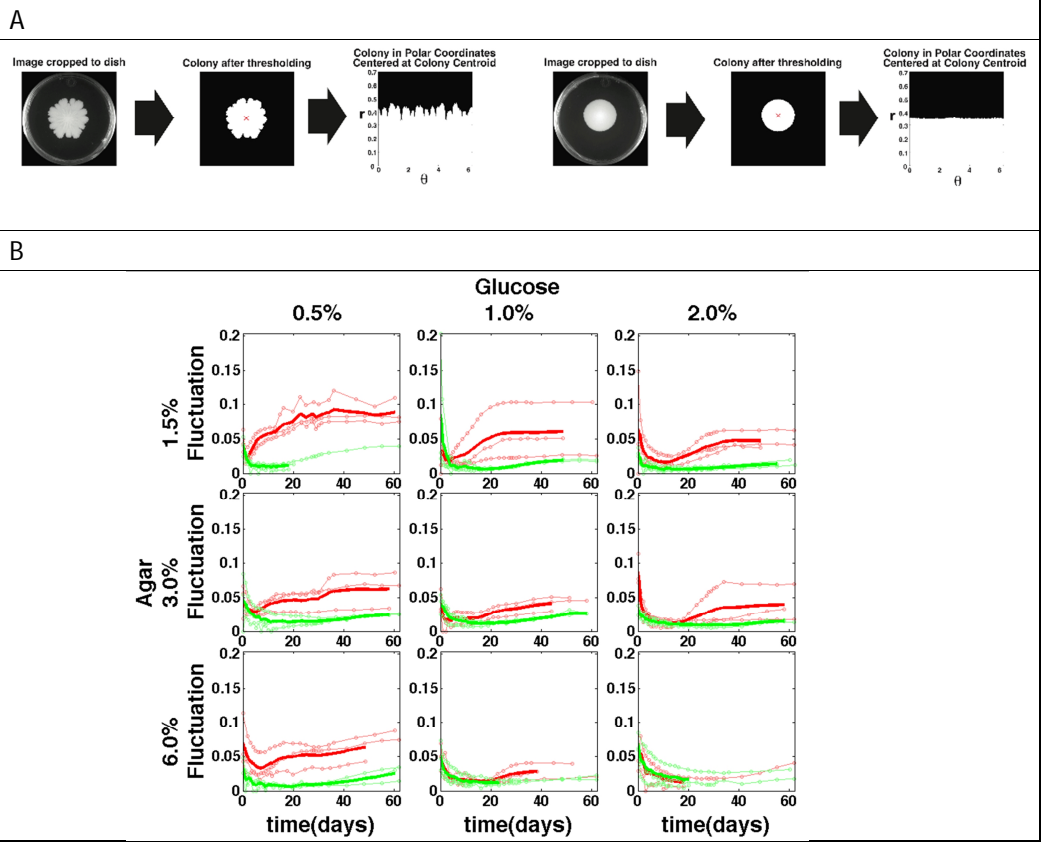

Supporting Figure S5.

Supplement: S5 Figure — Colony irregularity measured by Boundary Fluctuation is higher for FLO11 cells compared to flo11Δ cells. (A) Images of FLO11 and flo11Δ colonies were segmented and the boundaries were quantitatively analyzed in polar coordinates. (B) The boundary fluctuation of FLO11 (red curves) was inversely increasing with both agar and glucose concentrations. Minimal fluctuation was observed at the colony boundaries of flo11Δ mutants (green curves), compared to the conspicuous fluctuation at the FLO11 boundaries (red curves). Thinner curves were different replicates and thicker curves were their average up to a time when all the replicates were present. (PDF) [file pcbi.1003979.s005.pdf]

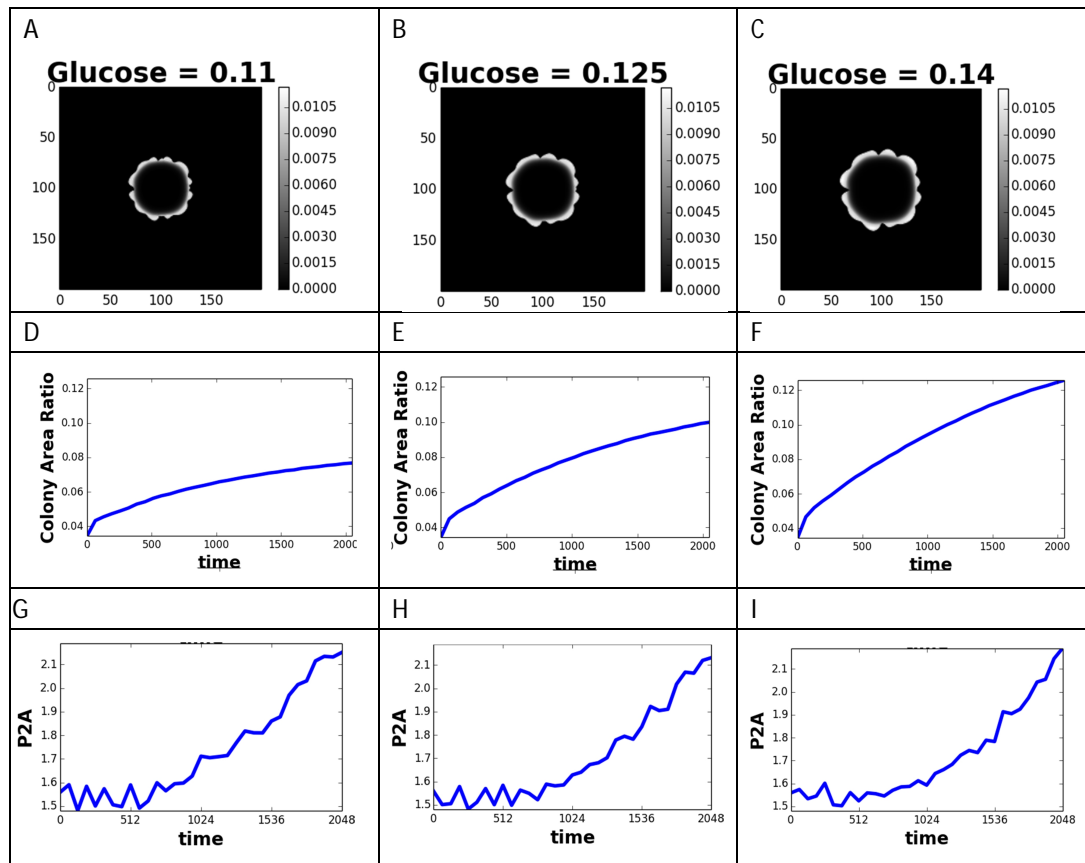

**Supporting Figure S6.**

Supplement: S6 Figure — Mathematical model of flo11Δ colony expansion. (A–C) Snapshots of colonies from simulations with a phenomenological third dimension (modeled by inclusion of a cell density-dependent escape term). The images show cell density at the agar surface at the end of a simulation for three different initial glucose levels. Introducing the escape term caused smaller colony size compared to simulations of FLO11 colonies without the escape term ( Fig. 2 ). (D–F) Time-courses of colony area (scaled to simulation box) for the three glucose conditions indicated. The maximum and overall area was higher for higher glucose concentrations, in agreement with the experimental results in Fig. 1. (G–I) Time-courses of P2A for the three glucose conditions. (PDF) [file pcbi.1003979.s006.pdf]

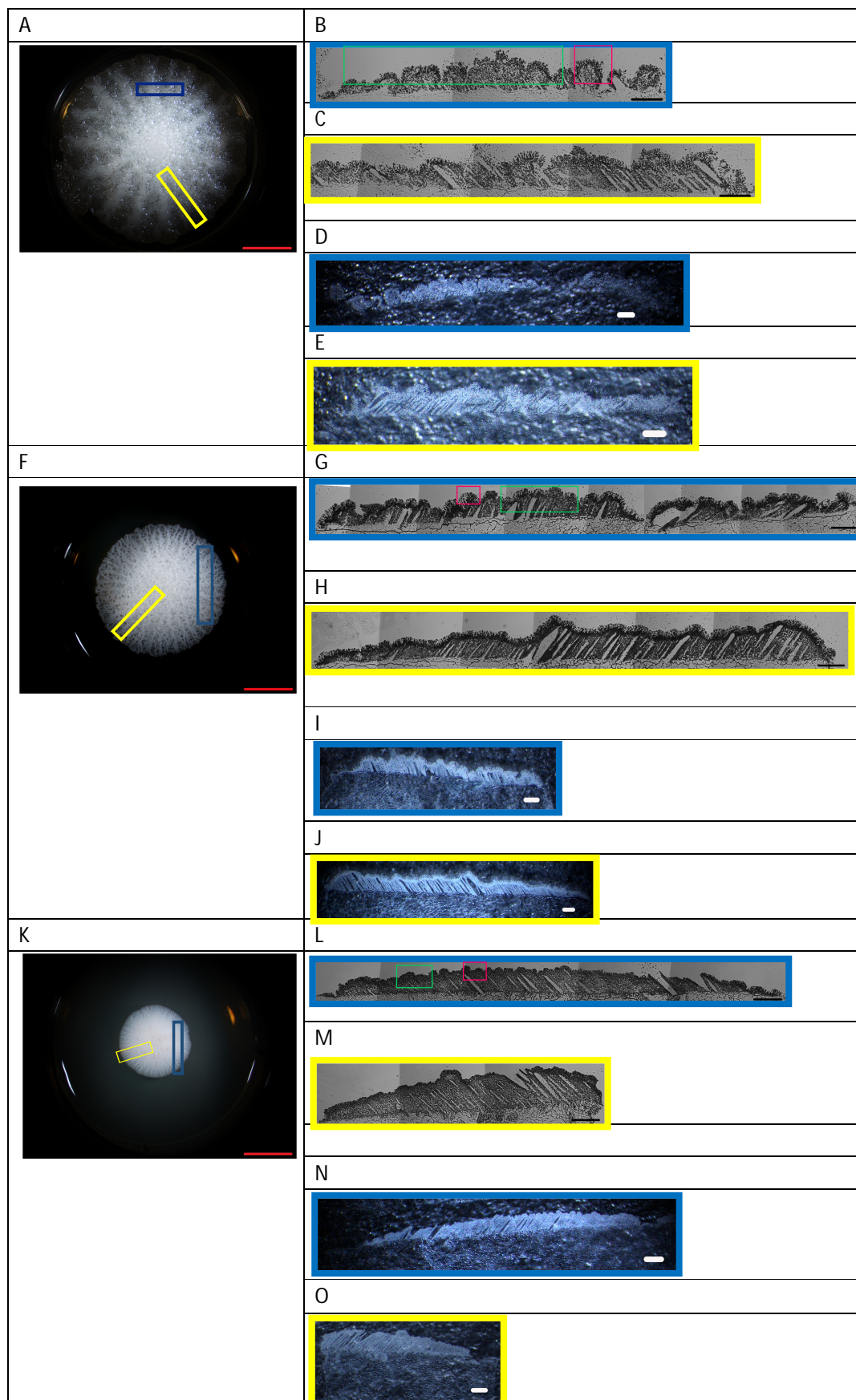

**Supporting Figure S7.**

Supplement: S7 Figure — The cross-sectional view of the hierarchical wrinkles of FLO11 colony expanding at various agar concentrations. (A, F, K) FLO11 colonies on 0.6%, 1.5%, or 3.0% YPD agar plates were cryosectioned at across-spokes (blue indicated estimated location) and radial (yellow indicated estimated location) orientations. Scale bars were 7.5 mm. (B, D, G, I, L, N) On 0.6%, 1.5%, or 3.0% agar, the cross-sectional view of the across-spokes (blue) oriented section from FLO11 colonies, two degrees of wrinkling composed of shorter-wavelength wrinkles at the surface of the colony (red box) and the longer-wavelength spokes underneath it (green box). (C, E, H, J, M, O) On 0.6%, 1.5%, or 3.0% YPD agar plates, only shorter-wavelength wrinkles were shown on the radial (yellow) cross-sectional view of the FLO11 colonies. (B, C, G, H, L, M) Images were taken under Nikon Eclipse TE2000-E Microscope, (D, E, I, J, N, O) Images were taken under Leica MZ6 stereo microscope (See Methods). Overall, the hierarchical wrinkles were observed on FLO11 colonies on various agar conditions. (B–E, G–J, L–O) Scale bar were 500 µm. (PDF) [file pcbi.1003979.s007.pdf]

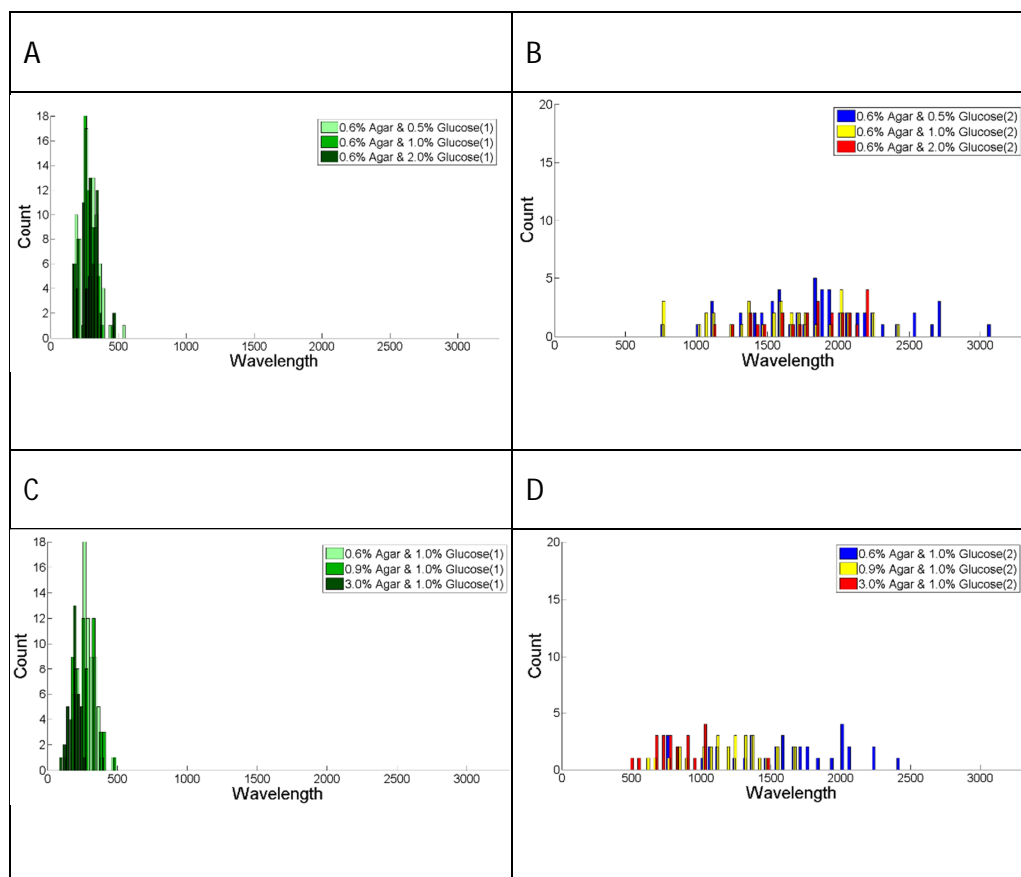

**Supporting Figure S8.**

Supplement: S8 Figure — The distribution of inter-spoke distances (wavelengths) of FLO11 colony depended inversely on agar density. (A, B) The wavelength distribution for both primary wrinkles (1) (A) and secondary wrinkles (spokes) (2) (B) were insensitive to the change of glucose concentrations. (C, D) The wavelength distribution for secondary wrinkles (spokes) (2) (D) shifted to shorter mean wavelengths with the increase in agar density. The wavelength distribution of the primary wrinkles (1) (C) was insensitive to the change of agar density. Primary and secondary wrinkles (spokes) were indicated by (1) and (2), respectively, in the figure legend. The unit for the wavelength in all panels was µm. (PDF) [file pcbi.1003979.s008.pdf]

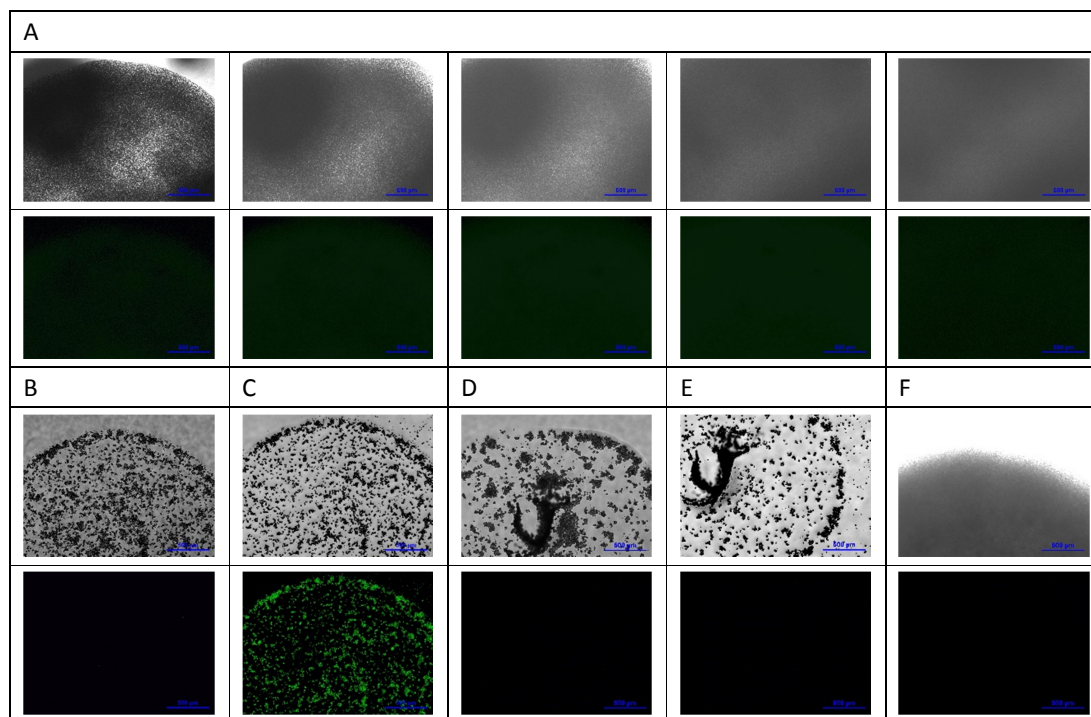

**Supporting Figure S9.**

Supplement: S9 Figure — Cell death was minimal and uniform during FLO11 colony expansion. (A) No particular death pattern has been detected on 0.6% agar and 1.0% glucose YPD plate with 5 µM Sytox green nucleic acid, at 19:40 hours, 26:20 hours, 30:50 hours, 41:40 hours and 67:40 hours after inoculation (images extracted from S4 and S5 Movies). (B–C) FLO11 colony incubated with 5 µM Sytox was imaged before (at 14 hours after inoculation) (B) and after adding of 3% H2O2 (at 15 hours after inoculation) (C) as positive control. Minimal cell death was detected (bottom panel) in the former, and uniform cell death was observed for the latter at 36 hours. (D–E) In the absence of Sytox as a negative control, no fluorescence was observed before (at 20 hours) (D) or after (at 42 hours) (E) adding 3% H2O2 at 21 hours. (F) No fluorescence was observed for FLO11 colony with neither Sytox nor H2O2. (A–F) Bright field and FITC channel were shown on the top and bottom panels respectively. (PDF) [file pcbi.1003979.s009.pdf]

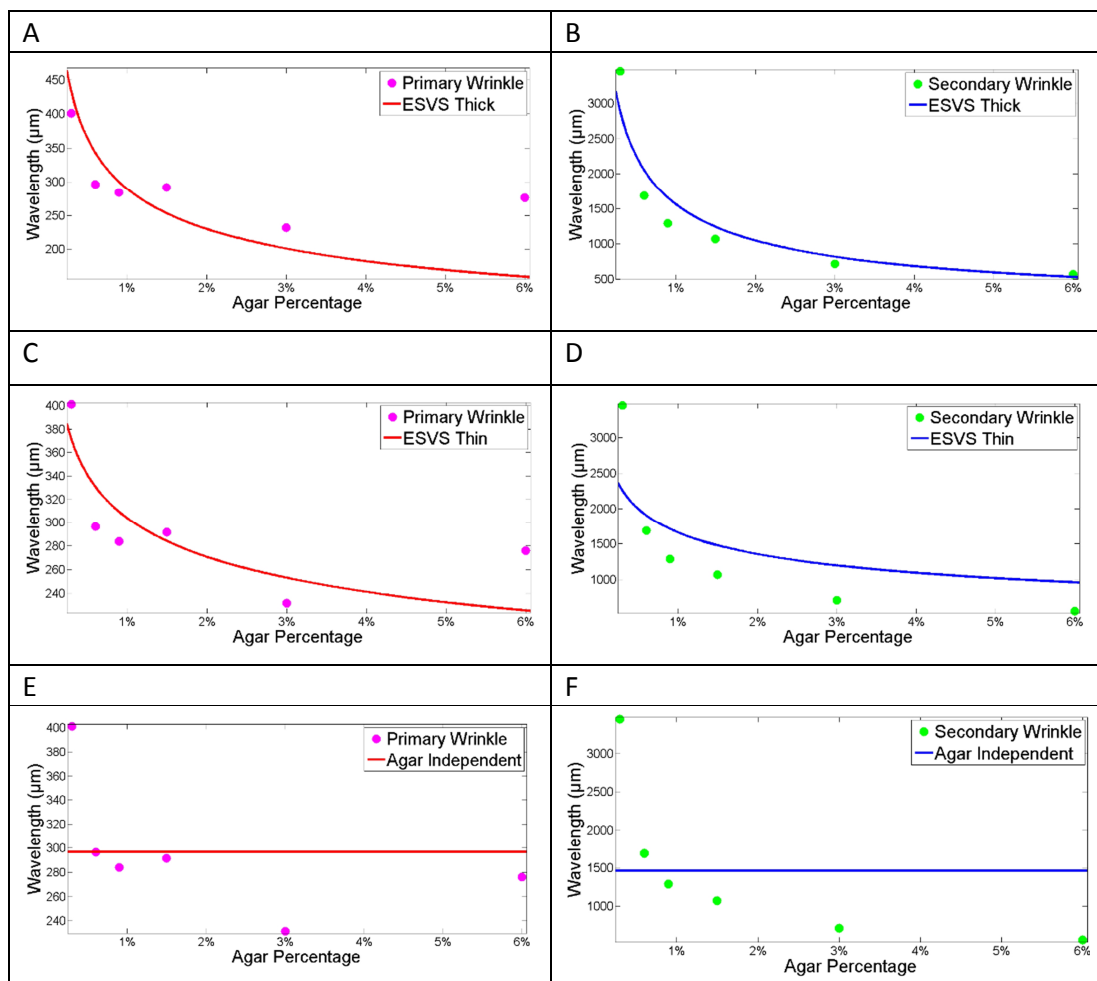

Supporting Figure S10.

Supplement: S10 Figure — ESVS thick and thin substrate model fits to experimentally measured wavelengths for primary and secondary wrinkles. The geometric mean wavelength of the primary (A, C, E) and secondary wrinkles (B, D, F) was plotted in magenta and green, respectively for FLO11 colonies growing on YPD media with various agar densities (0.3%, 0.6%, 0.9%, 1.5%, 3.0%, and 6.0%). (A, B) ESVS thick substrate model fits to primary (A) and secondary wrinkles (B) with R-square values of −0.23 and 0.89, respectively. (C, D) ESVS thin substrate model fit to primary (C) and secondary wrinkles (D) with R-square values of 0.63 and 0.6 respectively. (E, F) The agar-independent model fit both primary (E) and secondary wrinkles (F) with R-square values of 0. We obtained very similar results when using the arithmetic mean. (PDF) [file pcbi.1003979.s010.pdf]

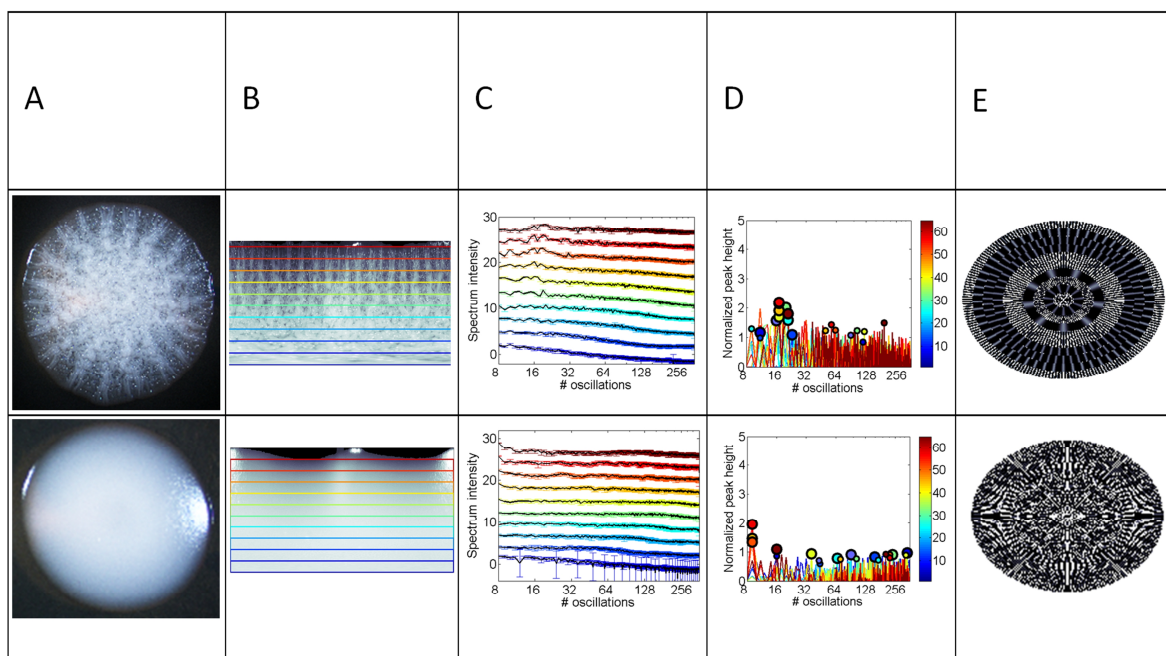

**Supporting Figure S11.**

Supplement: S11 Figure — Pipeline of FFT analysis for FLO11 and flo11Δ colonies. (A) Sample images of FLO11 (top) and flo11Δ (bottom) colonies used for analysis. (B) Original images were transformed into polar coordinates, and divided into 10 horizontal sections. (C) The mean spectra and the standard deviation of the spectra resulting from Fast Fourier Transformation analysis within each section. (D) Peak heights within the spectra were calculated based on t-test scores and the highest peaks were kept as the secondary and primary frequencies. Large circles marked the secondary frequencies, while smaller circles marked the primary frequencies. The top or bottom row corresponded to a FLO11 or flo11Δ colony. Both colonies were grown on 0.5% glucose, 0.9% agar YPD plates at 4 days. (E) FFT analysis processed images of FLO11 (top) and flo11Δ (bottom) colonies. (PDF) [file pcbi.1003979.s011.pdf]

|               |                                                                                     |                                                                                      |
|---------------|-------------------------------------------------------------------------------------|--------------------------------------------------------------------------------------|
|               | A                                                                                   |                                                                                      |
| <i>FLO11</i>  | 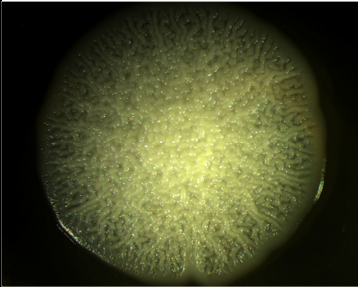   | 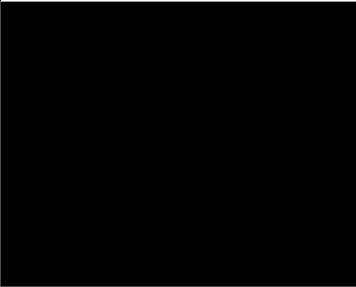   |
|               |                                                                                     |                                                                                      |
| <i>FLO11</i>  | B                                                                                   |                                                                                      |
|               | 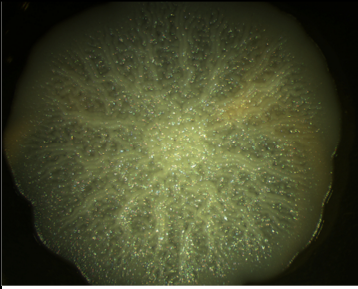   | 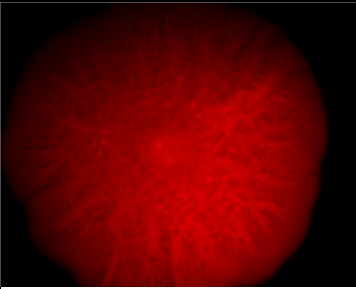   |
|               |                                                                                     |                                                                                      |
| <i>flo11Δ</i> | C                                                                                   |                                                                                      |
|               | 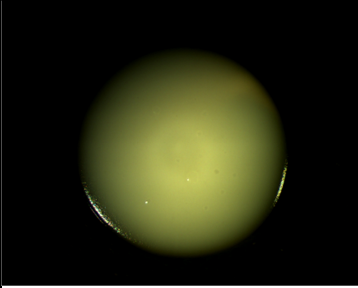  | 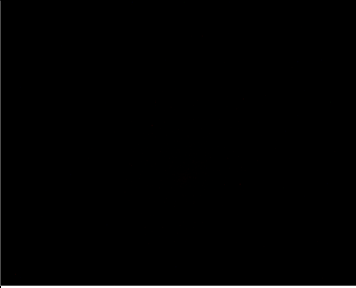  |
|               |                                                                                     |                                                                                      |
| <i>flo11Δ</i> | D                                                                                   |                                                                                      |
|               | 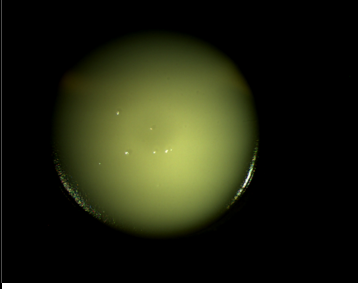 | 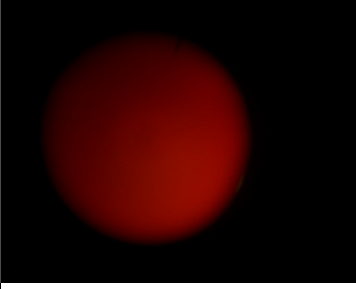 |
|               |                                                                                     |                                                                                      |

**Supporting Figure S12.**

Supplement: S12 Figure — The controls for competition between pattern forming FLO11 S. cerevisiae colonies and flo11Δ during head-to-head competition. (A) On 1.0% Agar YPGal plates with 0.5% galactose, unlabeled FLO11 expanded with regular pattern formation. (B) Pattern forming mCherry labeled FLO11 colony was visualized by mCherry fluorescence throughout the colony (right panel). (C) Unlabeled flo11Δ colony expanded without pattern. (D) mCherry labeled flo11Δ colony was visualized by mCherry fluorescence (right panel) with no pattern formation. Contrast is adjusted in photoshop for mCherry images. (PDF) [file pcbi.1003979.s012.pdf]

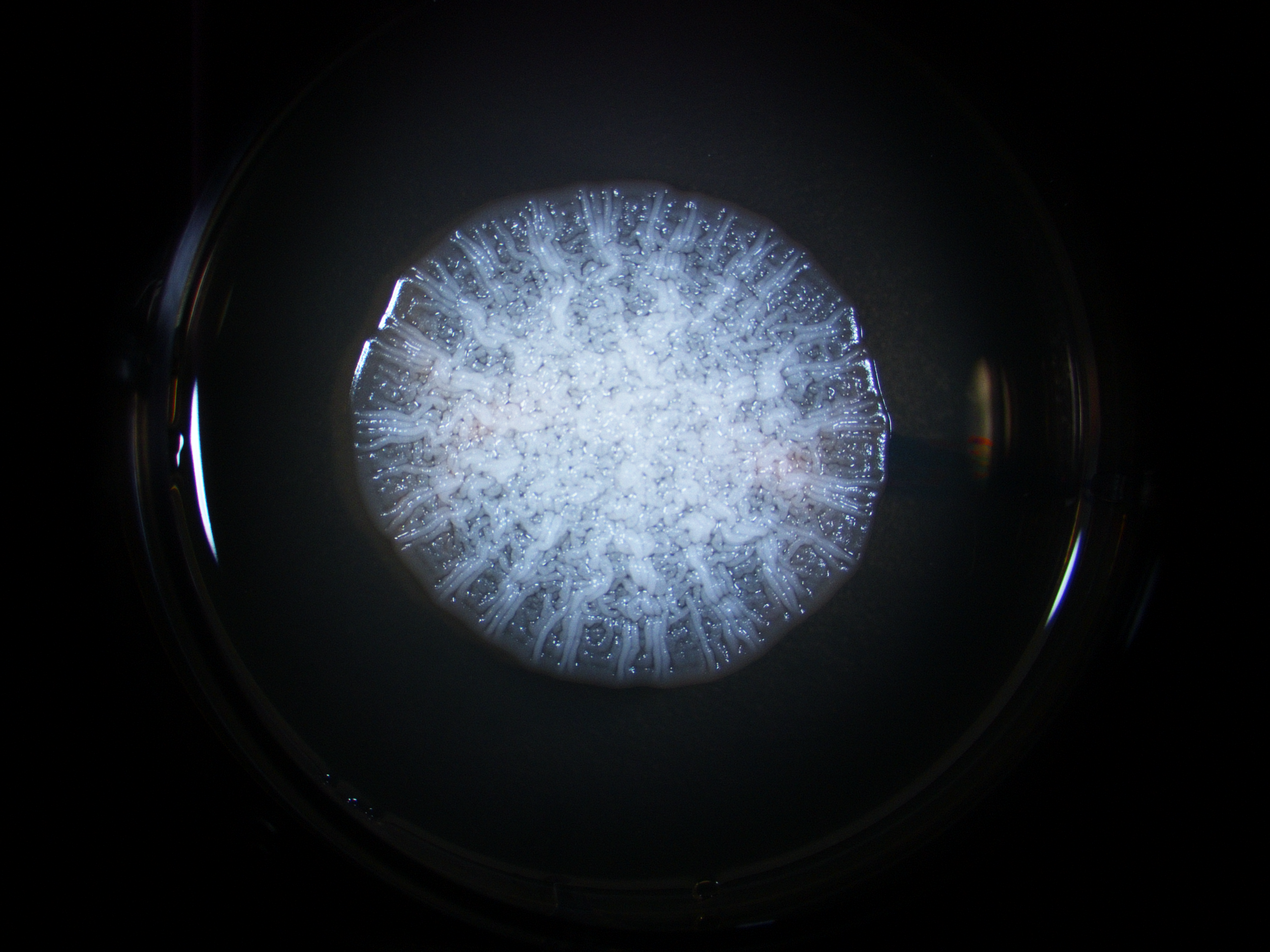

Supplement: S1 Dataset — This compressed file contains tab-delimited text data files and other documents in 5 subfolders: (i) Colony_Area&Irregularity contains colony area and irregularity values for various glucose and agar combinations (indicated by the file names, for example A15G05TBR1 means FLO11 colony in 1.5% agar and 0.5% glucose); (ii) PDEmodel_python contains the files for numerically solving the PDE model of colony expansion; (iii) WrinkleDistance_Detected contains tab-delimited data of colony surface patterns detected using FFT; (iv) WrinkleDistance_Measured contains tab-delimited data of inter-wrinkle distances (human measurement); (v) FFT contains an example for image segmentation followed by Fast Fourier Transformation to extract significant frequencies/wavelengths. (ZIP) [file pcbi.1003979.s021.zip › SuppData/FFT/Leica Flower plate-130219 tif/G05A09TBR1_R1_20130223_09hour_23_min.tif]

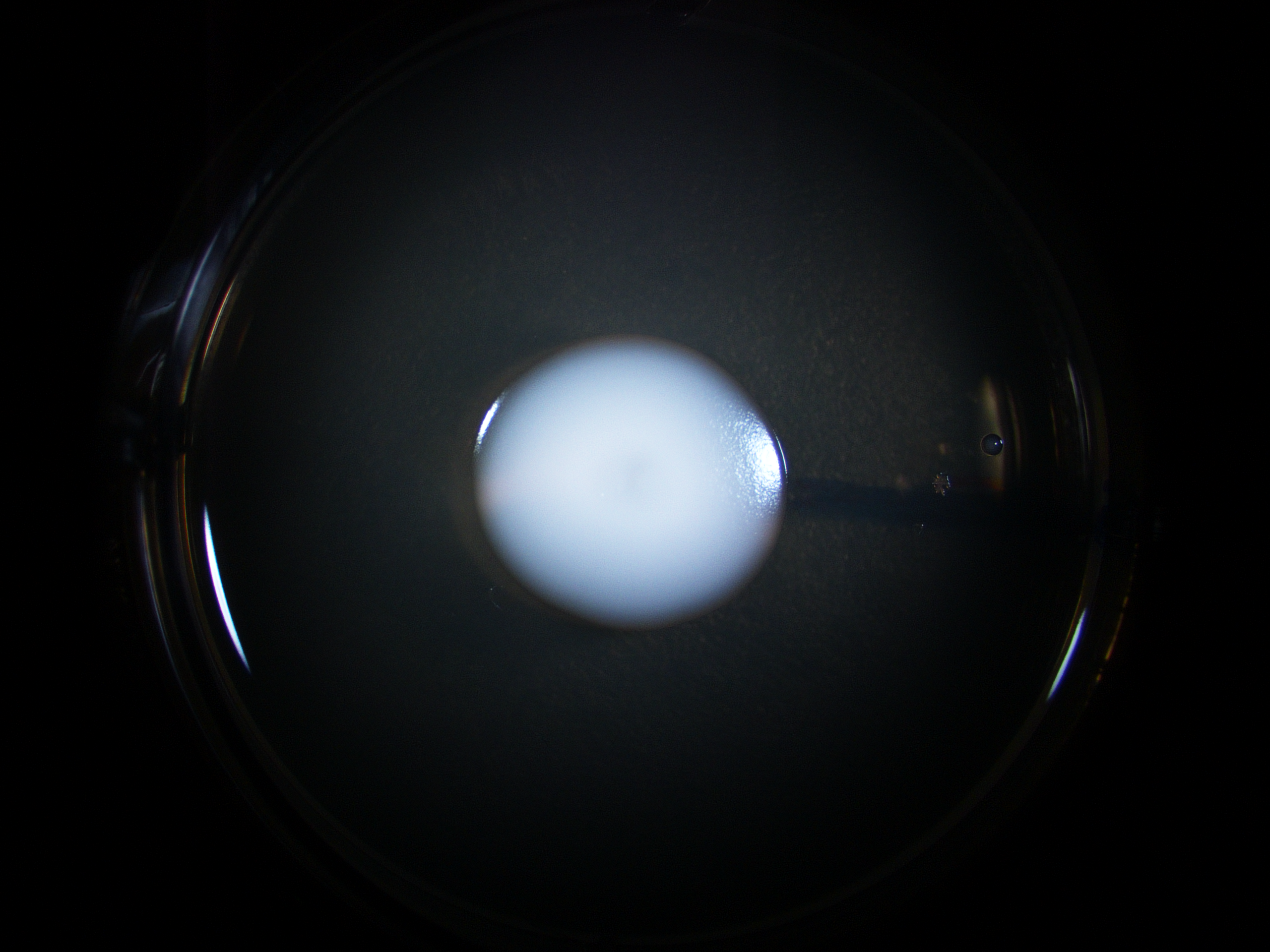

Supplement: S1 Dataset — This compressed file contains tab-delimited text data files and other documents in 5 subfolders: (i) Colony_Area&Irregularity contains colony area and irregularity values for various glucose and agar combinations (indicated by the file names, for example A15G05TBR1 means FLO11 colony in 1.5% agar and 0.5% glucose); (ii) PDEmodel_python contains the files for numerically solving the PDE model of colony expansion; (iii) WrinkleDistance_Detected contains tab-delimited data of colony surface patterns detected using FFT; (iv) WrinkleDistance_Measured contains tab-delimited data of inter-wrinkle distances (human measurement); (v) FFT contains an example for image segmentation followed by Fast Fourier Transformation to extract significant frequencies/wavelengths. (ZIP) [file pcbi.1003979.s021.zip › SuppData/FFT/Leica Flower plate-130219 tif/G05A09TBR5_R1_20130223_09hour_20_min.tif]
