# Supplementary material for: Two-Dimensionality of Yeast Colony Expansion Accompanied by Pattern Formation
Source: PLoS Comput Biol. 2014 Dec 11;10(12):e1003979. doi: 10.1371/journal.pcbi.1003979 (PMC4263361; doi:10.1371/journal.pcbi.1003979)
Supplement: S13 Figure — FLO11 S. cerevisiae cells out-expand flo11Δ cells during head-to-head competition. Replicate #2 of competing FLO11 and flo11Δ cells on 1.0% agar, 0.5% galactose YPGal plates. (A, B) Minimal competition between isogenic strains of unlabeled and mCherry labeled flo11Δ sectors. (C, D) Minimal competition was observed between unlabeled and mCherry labeled FLO11 sectors. (E, F) unlabeled FLO11 sector out-expanded mCherry labeled flo11Δ sectors with a conspicuous increase in the unlabeled sector angle. (G, H) Reverse labeling of (E, F). Bright field (A, C, E, G) and mCherry (B, D, F, H) were shown respectively. Contrast is adjusted in Adobe Photoshop CS for mCherry images. (PDF) [file pcbi.1003979.s013.pdf]

	A	B
flo11Δ vs.<br>*flo11Δ*	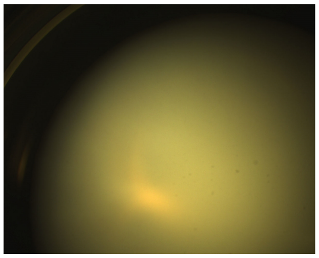	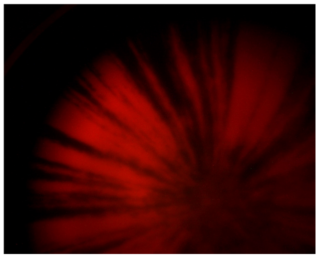
	C	D
FLO11 vs.<br>*FLO11*	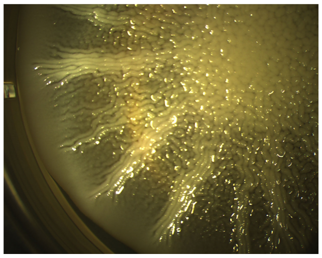	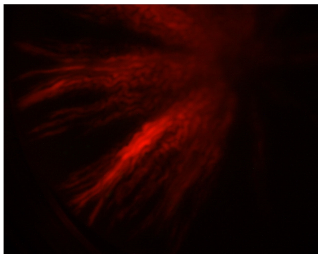
	E	F
FLO11 vs.<br>*flo11Δ*	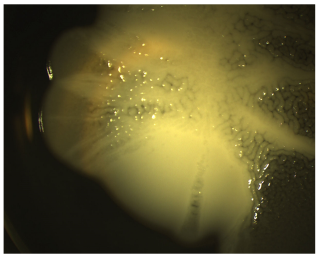	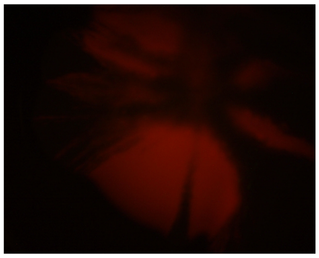
	G	H
flo11Δ vs.<br>*FLO11*	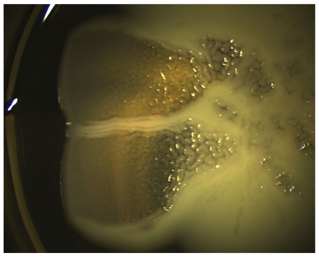	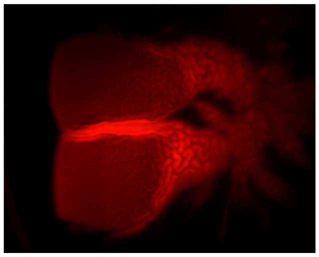

Supporting Figure S13.
