# Supplementary material for: Two-Dimensionality of Yeast Colony Expansion Accompanied by Pattern Formation
Source: PLoS Comput Biol. 2014 Dec 11;10(12):e1003979. doi: 10.1371/journal.pcbi.1003979 (PMC4263361; doi:10.1371/journal.pcbi.1003979)
Supplement: S1 Text — Supporting materials and methods. This document contains additional methods describing the procedures for recording the Supporting Movies. (PDF) [file pcbi.1003979.s022.pdf]

Supporting Information

Two-Dimensionality of Yeast Colony Expansion Accompanied by Pattern Formation

Lin Chen[1], Javad Noorbakhsh[2], Rhys M. Adams[1], Joseph Samaniego-Evans[2], Germaine Agollah[1], Dmitry Nevozhay[1,3], Jennie Kuzdzal-Fick[1], Pankaj Mehta[2] and Gábor Balázsi[1,4,5*]

[1]Department of Systems Biology – Unit 950, 7435 Fannin Street, The University of Texas MD Anderson Cancer Center, Houston, TX 77054, USA

[2]Department of Physics, Metcalf Science Center (SCI), Boston University, 590 Commonwealth Ave, Boston, MA 02215, USA

[3]School of Biomedicine, Far Eastern Federal University, 8 Sukhanova Street, Vladivostok, 690950, Russia

[4]Laufer Center for Physical & Quantitative Biology Rm 115C, Laufer Center, Z-5252, Stony Brook University, Stony Brook, NY 11794, USA

[5]Department of Biomedical Engineering, Stony Brook University, Stony Brook, NY 11794, USA

1. Supporting Materials and Methods

1.1 Visualizing the death pattern in *S. cerevisae* colonies. 5 μM Sytox (SYTOXR Green Nucleic Acid Stain, Life Technologies) was added to the 0.6% agar and 1.0% glucose YPD plate to detect the viability of cells during pattern formation. A time-lapse movie examining cell death in a *FLO11* colony was created by imaging under Nikon Eclipse Ti microscope with FITC filter and bright field, every 10 minutes for 72 hours. The bright field and FITC channel of the movie were obtained at 22 FPS and 15 FPS, accelerated by 32 times respectively (Supporting Movie 2). The color depth of the movie in FITC channel (Supporting Movie 2) was reduced to 8 bit via *rgb2ind* in Matlab. The negative controls were *FLO11* colonies grown on YPD plate without Sytox, in the presence (before (Supporting Figure 8D) or after (Supporting Figure 8E) adding hydrogen peroxide) or absence (Supporting Figure 8F) of 3% hydrogen peroxide. The positive control was YPD plate with 5μM Sytox and 3% hydrogen peroxide added by 15 hours (before (Supporting Figure 8B) or after (Supporting Figure 8C) adding hydrogen peroxide). The contrast was adjusted by the same threshold for experimental images, movies and controls.

1.2 Time lapse movies of pattern formation on the colony surface. 0.5 μl of *FLO11* (OD_{600} at 0.245) was inoculated into a 6 cm diameter YPD plate (0.6% agar and 1.0% glucose), and incubated at 30ºC (Barnstead Lab-Line stationary incubator). A robot built with a Lego Mindstorms NXT kit inverted the base of the plate and imaged with a Canon SD850 IS camera once an hour over 10 days. All images were aligned using Evangelidis' ECC image alignment algorithm [1]. Plates were identified and cropped using the Hough circle detection algorithm [2]. Images were compiled into an AVI sequence using ImageJ [3]. The movie was accelerated by 8 times.

1.3 Time lapse movies of head-to-head competition: The mixture of unlabeled *FLO11* and mCherry-labeled *flo11*Δ cells was inoculated on 0.6% Agar, 1.0% galactose YPGal plates and imaged using a Nikon Eclipse Ti microscope every 10 minutes for 30 hours to generate the movie on competition (Supporting Movie 3). The movie was contrast-adjusted with the same threshold as the control images, and accelerated by 8 times (Supporting Movie 3).

1.4 **Growth rate measurement for *FLO11* and *flo11Δ*.** *FLO11* and *flo11Δ* cells were inoculated in Yeast Extract Peptone Galactose (YPGal) media with 0.5% galactose, at the same starting OD_{600} = 0.005. Three independent replicates were initiated from three different *FLO11* and *flo11Δ* colonies. OD_{600} was measured at 2- to 4- hour intervals for the 57 hour assay while the cultures were incubated in a 311DS LabNet shaking Incubator. The size distribution of the *FLO11* and *flo11Δ* cells was affected by clumping due to incomplete separation at cell division. *FLO11* and *flo11Δ* clumps and single cells were distinguished by thresholding the diameters of objects obtained in Nexcelom CBA version 2.1.4.2. We exported and then loaded the data into Matlab for plotting.

1.5 *FLO11* and *flo11Δ* expansion movie. *FLO11* and *flo11Δ* cells were inoculated to the Yeast Extract Peptone Galactose (YPGal) media at 6ml in each well of the 6-well plate (BD Falcon, Cat#: 353046) with 0.5% galactose and 1.0% agar, at the same starting OD_{600} = 0.001. The initial colony expansion was imaged under a Nikon Tie microscope every 10 minutes for 26 hours.

1.6 Fitting of various physical models to manual measured wavelengths. We considered the two limiting cases that the ESVS system theory can capture: (i) when the substrate is very thick or infinite (ESVS thick) and (ii) when the substrate is thin, its height being comparable to the skin on its surface (ESVS thin). ESVS thin and ESVS thick models were fitted to the manual measured wavelengths for various concentrations of agar separately for the primary wrinkles and secondary

wrinkles (spokes) keeping one to three parameters free, such as the yeast Young's Modulus, or the coefficients a and b defining the dependence of agar Young Modulus on agar density (% agar). We reasoned that a and b must be unknown for the primary wrinkles, but the yeast Young modulus must be known since they involve the top layer of yeast cells on top of the matrix. On the contrary, a and b must be known while the skin Young modulus is unknown for the secondary wrinkles since they involve the primary-wrinkled skin on top of the agar, of known Young's Modulus.

The ESVS thick model fitting results were (with 95% confidence bounds):

$80(\mu m) \times \sqrt[3]{\frac{112(\mathrm{kPa})}{(\mathrm{a}x^2+\mathrm{b}x)}}, a, b > 0$ (primary wrinkles)

a = 0.0007498 (-8.756e+06, 8.756e+06)
b = 236.2 (-7.26, 479.7)

Goodness of fit: SSE=1.947e+04; R-square=-0.2375<0; RMSE=69.76

$325(\mu m) \times \sqrt[3]{\frac{\mathrm{YM\_Colony}}{(798500x^2+3923x)}}$, YM_Colony>0 (secondary wrinkles)

YM_Colony = 1.321e+04 (4583, 2.184e+04)

Goodness of fit: SSE=6.362e+05; R-square=0.8856; RMSE=356.7

The ESVS thin model was adopted according to [4,5]:

$\lambda = (hH)^{\frac{1}{2}}(\frac{E_m}{E_p})^{\frac{1}{6}}$

When we include the measured height of the yeast biofilm, yeast Young's Modulus and agar the fitting results were (with 95% confidence bounds):

$\Lambda = (80(\mu m) \times H(\mu m))^{\frac{1}{2}} \times \sqrt[6]{\frac{112(\mathrm{kPa})}{(ax^2+ax)}}$ (primary wrinkles)

a = 0.0001576 (-6.123e+05, 6.123e+05)
b = 3.735 (-0.6168, 8.087)

Goodness of fit: SSE=5761; R-square=0.6337; RMSE=37.95

$\Lambda = (325(\mu m) \times 6236(\mu m))^{\frac{1}{2}} \times \sqrt[6]{\frac{\text{YM\_Colony}}{(798500x^2+3923x)}}$ (secondary wrinkles)

Y = 297.8 (-463.8, 1059)

Goodness of fit: SSE=2.222e+06; R-square=0.6006; RMSE=666.6

In the above fits, the height of the agar $6236(\mu m)$ was calculated by

$$V_{\text{Agar}} = \pi R^2 H$$

where R is the radius (1.75 cm) of the 6-well plate (BD Falcon, Cat#: 353046), V_{Agar} is the volume of the agar (6 ml). In addition, we considered a trivial agar-independent model, for which we obtained R^2=0, as expected. Young's modulus for the top cell layer (producing primary wrinkles) was varied between two extremes: ~20 Pa and 122 MPa. The lower of these values (20 Pa) corresponds to the Young's Modulus of bacterial biofilms [6]. The higher of these values (122 MPa) corresponds to the Young's Modulus of single yeast cells [7]. While the best-fit parameters depended on the value of the Young's Modulus, we robustly obtained the same quality of fits for the ESVS thin and thck models, with identical R-square across fits, regardless of this value. The fitting was performed with the Matlab curve fitting tool called *cftool*.

Modeling escape into the 3rd (vertical) direction

The 3D model for *flo11Δ* was constructed by implicit inclusion of the z-coordinate through an escape (sink) term. We assumed that the likelihood of vertical escape increases with cell density, contributing to an increase in the height of the colony. The equations for this model were:

$$\frac{d\rho(x,y,t)}{dt} = \nabla.\left(D_\rho \nabla\rho(x,y,t)\right) + \alpha g(x,y,t)\rho(x,y,t) - \gamma\rho(x,y,t)$$

$$\frac{dg(x,y,t)}{dt} = D_g\nabla^2 g(x,y,t) - \beta g(x,y,t)\rho(x,y,t)$$

$$\frac{dz(x,y,t)}{dt} = \gamma\rho(x,y,t)$$

$$D_\rho = \rho\Theta(\rho - \rho_0)\Theta(g - g_0)$$

These equations were similar to the equations proposed for *FLO11* colony growth with the addition of the escape term $-\gamma\rho(x,y,t)$ describing cell movement into the z-direction. Furthermore the changes in the height of the colony had been modeled by an independent equation for z. All of the parameters were identical to the *FLO11* equations, and we chose $\gamma = 0.05$.

Supporting References

1. Evangelidis GD, Psarakis EZ (2008) Parametric image alignment using enhanced correlation coefficient maximization. IEEE Trans Pattern Anal Mach Intell 30: 1858-1865.
2. Ballard DH (1981) Generalizing the Hough Transform to Detect Arbitrary Shapes. Pattern Recognition 13: 111-122.
3. Schneider CA, Rasband WS, Eliceiri KW (2012) NIH Image to ImageJ: 25 years of image analysis. Nature Methods 9: 671-675.
4. Vandeparre H, Pineirua M, Brau F, Roman B, Bico J, et al. (2011) Wrinkling hierarchy in constrained thin sheets from suspended graphene to curtains. Phys Rev Lett 106: 224301.
5. Vandeparre H, Damman P (2008) Wrinkling of stimuloresponsive surfaces: mechanical instability coupled to diffusion. Phys Rev Lett 101: 124301.
6. Lau PCY, Dutcher JR, Beveridge TJ, Lam JS (2009) Absolute Quantitation of Bacterial Biofilm Adhesion and Viscoelasticity by Microbead Force Spectroscopy. Biophysical Journal 96: 2935-2948.
7. Smith AE, Zhang ZB, Thomas CR, Moxham KE, Middelberg APJ (2000) The mechanical properties of Saccharomyces cerevisiae. Proceedings of the National Academy of Sciences of the United States of America 97: 9871-9874.
